# Hydrogen sulfide production in the medullary respiratory center modulates the neural circuit for respiratory pattern and rhythm generations

**DOI:** 10.1038/s41598-023-47280-9

**Published:** 2023-12-04

**Authors:** Minako Okazaki, Masayuki Matsumoto, Tadachika Koganezawa

**Affiliations:** 1 Department of Neurophysiology, Division of Biomedical Science, Institute of Medicine, University of Tsukuba, Tsukuba, Ibaraki 305-8575 Japan; 2 Doctoral Program in Neuroscience, Graduate School of Comprehensive Human Sciences, University of Tsukuba, Tsukuba, Ibaraki 305-8575 Japan; 3 Department of Cognitive and Behavioral Neuroscience, Division of Biomedical Science, Institute of Medicine, University of Tsukuba, Tsukuba, Ibaraki 305-8575 Japan; 4 Transborder Medical Research Center, University of Tsukuba, Tsukuba, Ibaraki 305-8575 Japan

**Keywords:** Respiration, Central pattern generators

## Abstract

Hydrogen sulfide (H_2_S), which is synthesized in the brain, modulates the neural network. Recently, the importance of H_2_S in respiratory central pattern generation has been recognized, yet the function of H_2_S in the medullary respiratory network remains poorly understood. Here, to evaluate the functional roles of H_2_S in the medullary respiratory network, the Bötzinger complex (BötC), the pre-Bötzinger complex (preBötC), and the rostral ventral respiratory group (rVRG), we observed the effects of inhibition of H_2_S synthesis at each region on the respiratory pattern by using an in situ arterially perfused preparation of decerebrated male rats. After microinjection of an H_2_S synthase inhibitor, cystathionine β-synthase, into the BötC or preBötC, the amplitude of the inspiratory burst decreased and the respiratory frequency increased according to shorter expiration and inspiration, respectively. These alterations were abolished or attenuated in the presence of a blocker of excitatory synaptic transmission. On the other hand, after microinjection of the H_2_S synthase inhibitor into the rVRG, the amplitude of the inspiratory burst was attenuated, and the respiratory frequency decreased, which was the opposite effect to those obtained by blockade of inhibitory synaptic transmission at the rVRG. These results suggest that H_2_S synthesized in the BötC and preBötC functions to limit respiratory frequency by sustaining the respiratory phase and to maintain the power of inspiration. In contrast, H_2_S synthesized in the rVRG functions to promote respiratory frequency by modulating the interval of inspiration and to maintain the power of inspiration. The underlying mechanism might facilitate excitatory synaptic transmission and/or attenuate inhibitory synaptic transmission.

## Introduction

Hydrogen sulfide (H_2_S) is an endogenous gas synthesized in the mammalian body, including in the brain^1^. In the central nervous system, it functions as a neuromodulator through presynaptic and postsynaptic mechanisms^2–6^. It can modify the release of neurotransmitters at the presynaptic terminal, as well as the postsynaptic properties by altering the activity of ion channels or receptors. These neuromodulatory effects indicate the potential roles of H_2_S in various physiologic systems based on the neural network. Some studies have suggested that H_2_S is involved in respiratory central pattern generation in the brainstem. Manipulation of H_2_S synthesis modified the respiratory frequency, inspiratory burst power, and respiratory pattern in in vitro experiments on medullary slices, in vivo experiments on anesthetized rats, and an in situ arterially perfused preparation^3,7,8^. H_2_S is also involved in ventilatory responses under hypoxia/hypercapnia conditions^8–10^. Although these results show the essential roles of H_2_S in respiration, the exact role of H_2_S in the respiratory network remains unknown.

A rhythmic respiratory pattern is generated at the respiratory network in the brainstem and spinal cord^11,12^. On the basis of respiratory phase-locked neural activities and functions in respiratory output, the medullary respiratory network is composed of some sub-regions including the pre-Bötzinger complex (preBötC), the Bötzinger complex (BötC), and the ventral respiratory group (VRG). The preBötC and BötC are known as pattern generators and kernel regions generating inspiration and expiration, respectively^13,14^. On the other hand, the rostral VRG (rVRG) contains inspiratory premotor neurons generating diaphragmic activity. Given the heterogeneity of the medullary respiratory network, the functional roles of H_2_S in respiratory pattern generation at each region need to be clarified for better understanding.

In this study, we aimed to reveal the respective functional roles in respiration of H_2_S synthesized in the BötC, preBötC, and rVRG. Here, we show that the inspiratory burst decreased and the respiratory pattern changed as a result of inhibition of H_2_S synthesis at the BötC, preBötC, or rVRG in a region-dependent manner. Moreover, the data indicate that the underlying mechanism might regulate excitatory or inhibitory synaptic transmission by H_2_S. We propose that H_2_S in the medullary respiratory network maintains respiratory rhythm and pattern through different regional mechanisms and functions.

## Methods

All procedures were performed according to the guidelines of the Animal Care and Use Committee of the University of Tsukuba and the ARRIVE guidelines and approved by the ethics review committee of the University of Tsukuba (permission numbers: 20-019, 21-030, and 22-0771).

### In situ arterially perfused preparation

Experiments were performed on 90 male Wistar rats (aged 3–4 weeks; Japan SLC, Inc.) using an in situ arterially perfused preparation. The general methods were based on those of previous studies^7,15^.

Briefly, the rats were intraperitoneally injected with heparin (100 U) and anesthetized deeply through isoflurane inhalation until respiration stopped. The rats were bisected subdiaphragmatically, and the digestive tract and lungs were removed and quickly transferred into iced artificial cerebrospinal fluid (ACSF). The composition of the ACSF was as follows (mM): NaCl (125), NaHCO_3_ (25), KCl (5), CaCl_2_ (2.5), MgSO_4_ (1.25), KH_2_PO_4_ (1.25), and D-glucose (10). The cerebrocortex and cerebellum were removed to expose the dorsal surface of the medulla. The descending aorta, left phrenic nerve, and left vagus nerve were isolated. After being transferred to the recording chamber, the descending aorta was cannulated with a double-lumen catheter, and the rats were perfused by use of a peristaltic pump (520U; Watson-Marlos Inc.) with ACSF containing 1.25% polyethylene glycol to maintain the oncotic pressure, gassed with 95% O_2_/5% CO_2_, and warmed to 29–31°C. The perfusion speed was kept constant on the basis of the rats’ body weight (13.0–29.1 mL/min). A vasoconstrictor peptide (arginine vasopressin, 0.4–1.6 nM) was added to the perfusate to increase peripheral vascular resistance. Neuromuscular paralysis was produced by vecuronium bromide (4 mg/L) to prevent electromyogram contamination. The perfusate leaked from the rats was collected by the recording chamber, re-gassed with 95% O_2_/5% CO_2_, and re-perfused from the descending aorta.

### Electrocardiography and peripheral nerve recording

The left phrenic and left central vagus nerve activities were recorded via glass suction electrodes. These activities were amplified (× 10,000) and filtered (100–5000 Hz). To obtain integrated waveforms of these nerve activities, they were rectified and smoothed by application of the moving average (0.05-s time window). The perfusion pressure (PP) was monitored via the second lumen of the double-lumen catheter. The electrocardiogram was recorded, and the heart rate (HR), calculated from the interval between two R waves.

### Microinjection of drugs into the medulla

Drugs were locally applied into the unilateral ventral respiratory column of the medulla via pressure injection from triple-barreled glass micropipettes. For the pressure injection, a pressure control valve and a solenoid valve of a custom-made apparatus regulated the pressure and duration of compressed air from a gas cylinder. The ventral respiratory column was first targeted stereotaxically, according to the obex. L-glutamate (L-glu, 10 mM, 30 nL) was microinjected around the following coordinates relative to the obex: 1.5 mm rostral to the obex, 1.6 mm lateral to the midline, and 2.5 mm ventral to the dorsal surface of the medulla. On the basis of the respiratory response to the L-glu microinjection, we identified the respiratory network^16,17^. L-glu injection into the preBötC increased respiratory frequency, whereas L-glu injection into the BötC decreased it (Supplementary Fig. S1). Stable nerve activities were recorded at least 200 s after insertion of the pipette into the target region. In the H_2_S synthase (cystathionine β-synthase [CBS])-inhibiting experiments, hydroxylamine hydrochloride (HA; CBS inhibitor, 250 mM, 25 nL) was locally microinjected into the BötC (n = 39), preBötC (n = 43), or rVRG (n = 28). We used the same rats for injecting drugs into another region in cases of restored respiratory patterns and nerve activities after HA injection. In the experiments with blockade of synaptic transmission, kynurenic acid (KYN, 50 mM, 25 nL) or a cocktail of bicuculline and strychnine (B + S, 250 μM each, 25 nL) were microinjected as receptor blockers for excitatory and inhibitory transmissions, respectively. The concentration of those drugs was selected on the basis of previous reports^18–23^. All drugs were dissolved in saline except for KYN, which was dissolved in 0.1 M NaOH and diluted with saline or saline-containing pontamine sky blue. The pH was adjusted to pH7.4 by use of 0.1 M HCl. The microinjection of vehicle (saline, 25 nL) into the BötC, preBötC and rVRG did not affect the respiratory outputs (Supplementary Fig. S2).

### Histology

At the end of the experiment, dye (pontamine sky blue, 25 nL) was injected to mark the location of the injected site. The brainstem was removed and stored in a solution of 10% formalin. Coronal sections (50 μm) were cut and scanned to confirm the injected site on the basis of previous studies^11,16^. The ventral respiratory column was determined to be 1.6 to 2.2 mm lateral to the midline, which was ± 0.2 mm lateral to the center of the nucleus ambiguus. The depth of the injected sites was expressed as the distance from the ventral edge of the nucleus ambiguus. The rostrocaudal location was expressed as the distance from the caudal edge of the facial nucleus, and the caudal edge of the facial nucleus was determined as the rostral end of the BötC, which might include the parafacial respiratory column. Exceptions to these criteria were judged to be outside the preBötC, BötC, and rVRG. Furthermore, the ventral respiratory column was divided into the preBötC, BötC, and rVRG relative to the distance from the caudal edge of the facial nucleus (preBötC 0.1–0.5 mm caudal, BötC 0.6–1.0 mm caudal, rVRG 1.1–1.5 mm caudal, Supplementary Fig. S3).

### Data analysis

Outputs from the nerves and PP were recorded directly onto a computer hard drive through an AD converter (Power1401; Cambridge Electronic Design, Cambridge, UK) and analyzed using data capture and analysis software (Spike2; Cambridge Electronic Design, Cambridge, UK). To analyze the respiratory pattern from the phrenic nerve and vagus nerve activities, phrenic nerve burst-triggered averaging was applied in the integrated nerve recording. The waveform of the phrenic nerve burst-triggered averaging was obtained in a 20-s time window, and 10-s steps moved the time window to observe the temporal changes.

The amplitude of the nerve activities was measured from the averaged waveform. The duration of the inspiratory phase was determined by the duration from the onset of the phrenic nerve burst to the onset of the sharp decrease. The duration of a single respiratory cycle was measured from the offset of inspiration to the next offset of inspiration. The duration of the expiratory phase was calculated by subtraction of the inspiratory phase duration from the single respiratory cycle duration. The inspiratory ratio to the single respiratory cycle was calculated.

For standardization among the rats, the average of 100 s before the drug injection of the amplitude of nerve activities and the duration of each phase was determined as 100%, and these parameters after drug injection were expressed as the percentage change.

### Statistics

Numeric data were expressed as means ± SEMs. To compare two different groups statistically, the paired *t*-test or one-sample *t*-test was used. For multiple comparisons, we used ANOVA with repeated measures followed by the Dunnett test for comparison with the control. Significance levels were set as *p* < 0.05.

## Results

### Effects of H_2_S synthesis inhibition at the BötC

To evaluate the roles of endogenous H_2_S in the medullary respiratory network on the respiratory pattern, we locally microinjected HA into the BötC, preBötC, or rVRG in an in situ arterially perfused preparation.

**Figure 1:**
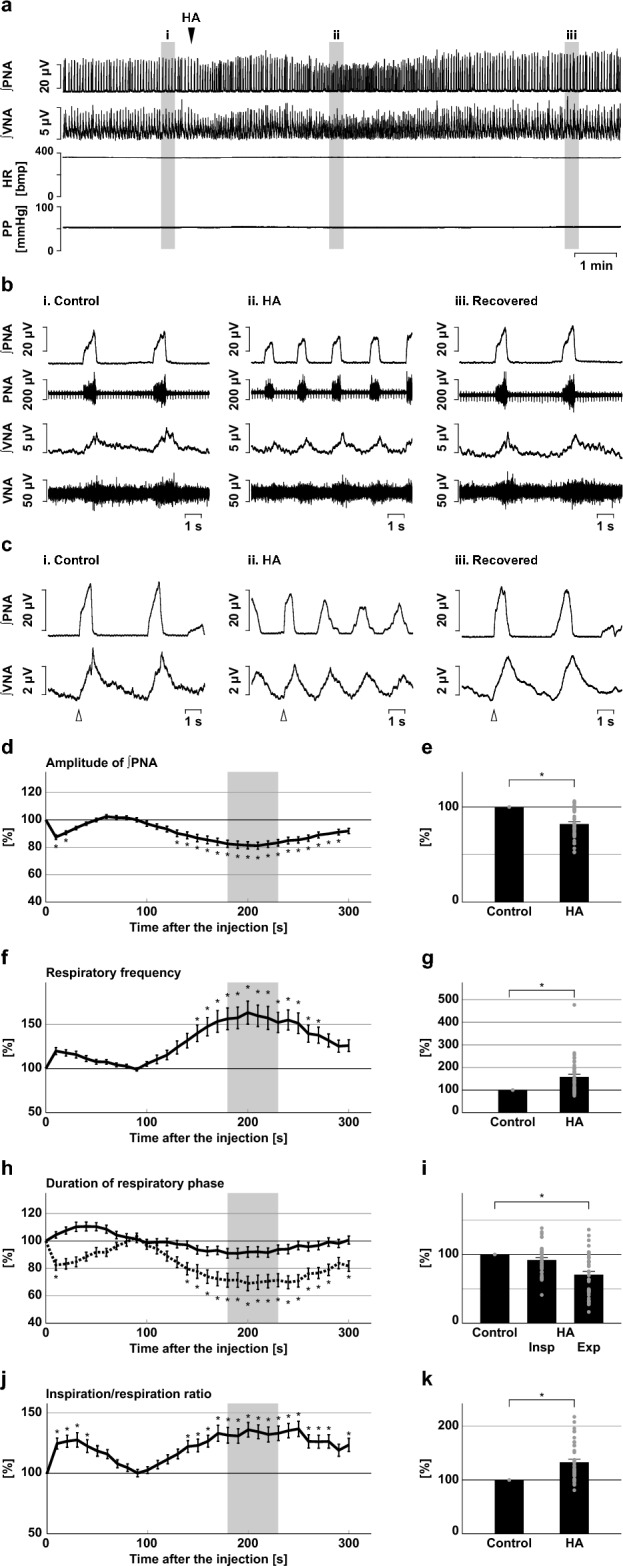
Effects of H_2_S synthesis inhibition at the BötC on respiration. (**a**) Overall effects of H_2_S synthesis inhibition produced by HA microinjection at the BötC on the integrated activities of the phrenic nerve (∫PNA) and vagus nerve (∫VNA), heart rate (HR), and perfusion pressure (PP). The triangle indicates the time of drug microinjection. (**b**) Raw waveforms of activities of the phrenic nerve (PNA) and vagus nerve (VNA), ∫PNA, and ∫VNA in the control (i), after HA microinjection (ii), and after recovery (iii). Each waveform was extracted from (i)–(iii) in (**a**). (**c**), Phrenic nerve burst-triggered averages of the ∫PNA and ∫VNA in the control (i), after HA microinjection (ii), and after recovery (iii). Each waveform was obtained by averaging the ∫PNA and ∫VNA during (i)–(iii) in (**a**). The triangle indicates the time of the trigger. (**d**), (**f**), (**h**), (**j**) Temporal changes in the amplitude of ∫PNA (**d**), the respiratory frequency (**f**), the duration of the inspiratory phase (**h**, solid line) and expiratory phase (**h**, dotted line), and the ratio of inspiration to respiration (**j**) (n = 39). (**e**), (**g**), (**i**), (**k**) The change rates of the amplitude of ∫PNA (**e**), the respiratory frequency (**g**), the duration of the inspiratory and expiratory phases (i), and the ratio of inspiration to respiration (**k**) after HA microinjection. The change rates were average in the gray areas in (**d**), (**f**), (**h**), and (**j**). The asterisks indicate *p* < 0.05 as compared with the control (one sample *t*-test in (**e**), (**g**), (**k**), Dunnett test in (**d**), (**f**), (**h**), (**i**), (**j**)). Results are expressed as means ± SEMs.

After injection of HA into the BötC, the amplitude of phrenic nerve activity and the respiratory frequency changed gradually and reached significant changes around 2 min after the HA injection (Fig. 1a–f). The amplitude of phrenic nerve activity significantly decreased (82.1 ± 2.5%, *p* < 0.001 vs. control; Fig. 1d,e). The amplitude of post-inspiratory vagus nerve activity significantly decreased (78.7 ± 4.0%, *p* < 0.001 vs. control; Supplementary Fig. S4a). The respiratory frequency significantly increased (157.8 ± 12.1%, *p* < 0.001 vs. control; Fig. 1f,g). These effects were gradually recovered and returned to the control levels within 10 min of the HA injection. To clarify the changes in the respiratory pattern, respiration was divided into inspiration and expiration, and the durations of these phases were measured. The duration of the expiratory phase significantly decreased (70.6 ± 4.9%, *p* < 0.001 vs. control), whilst the duration of the inspiratory phase did not change significantly (91.9 ± 3.4%, *p* = 0.174 vs. control; Fig. 1h,i). Therefore, the increasing respiratory frequency resulted from the shortening of the expiratory phase. Since the level of the effects on the inspiratory and expiratory phase durations was different, the ratio of inspiratory phase duration to respiration significantly increased (ratio of inspiratory phase in control, 0.22 ± 0.01, with HA, 0.28 ± 0.02, 132.9 ± 5.7%, *p* < 0.001 vs. control; Fig. 1j,k).

### Effects of H_2_S synthesis inhibition at the preBötC

**Figure 2:**
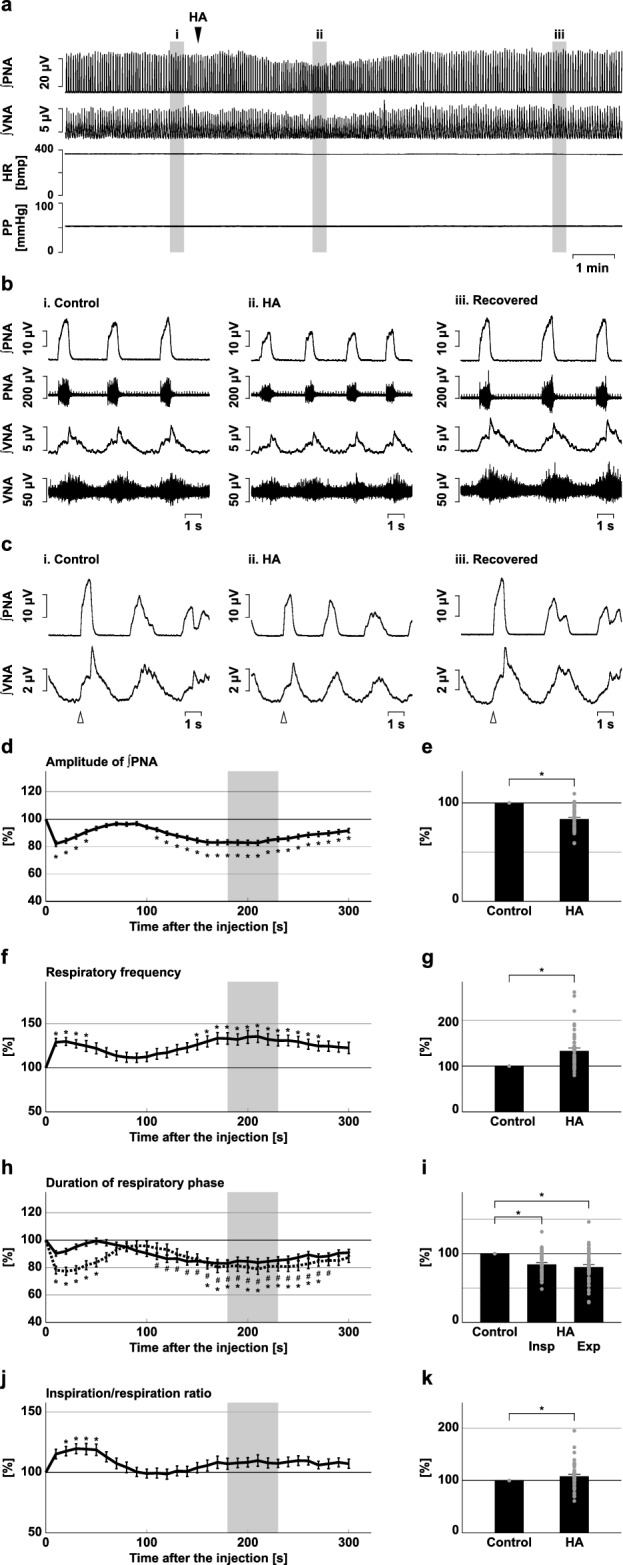
Effects of H_2_S synthesis inhibition at the preBötC on respiration. (**a**) Overall effects of H_2_S synthesis inhibition produced by HA microinjection at the preBötC on the integrated activities of the phrenic nerve (∫PNA) and vagus nerve (∫VNA), heart rate (HR), and perfusion pressure (PP). The triangle indicates the time of drug microinjection. (**b**) Raw waveforms of activities of the phrenic nerve (PNA) and vagus nerve (VNA), ∫PNA, and ∫VNA in the control (i), after HA microinjection (ii), and after recovery (iii). Each waveform was extracted from (i)–(iii) in (**a**). (**c**), Phrenic nerve burst-triggered averages of the ∫PNA and ∫VNA in the control (i), after HA microinjection (ii), and after recovery (iii). Each waveform was obtained by averaging the ∫PNA and ∫VNA during (i)–(iii) in (**a**). The triangle indicates the time of the trigger. (**d**), (**f**), (**h**), (**j**) Temporal changes in the amplitude of ∫PNA (**d**), the respiratory frequency (**f**), the duration of the inspiratory phase (**h**, solid line) and expiratory phase (**h**, dotted line), and the ratio of inspiration to respiration (**j**) (n = 43). (**e**), (**g**), (**i**), (**k**) The change rates of the amplitude of ∫PNA (**e**), the respiratory frequency (**g**), the duration of the inspiratory and expiratory phases (**i**), and the ratio of inspiration to respiration (**k**) after HA microinjection. The change rates were average in the gray areas in (**d**), (**f**), (**h**), and (**j**). The asterisks indicate *p* < 0.05 as compared with the control (one sample *t*-test in (**e**), (**g**), (**k**), Dunnett test in (**d**), (**f**), (**h**), (**i**), (**j**)). Results are expressed as means ± SEMs.

Next, we injected HA into the preBötC (Fig. 2). After microinjection of HA into the preBötC, the amplitude of phrenic nerve activity significantly decreased (83.6 ± 1.7%, *p* < 0.001 vs. control; Fig. 2d,e), the amplitude of post-inspiratory vagus nerve activity significantly decreased (79.1 ± 3.3%, *p* < 0.001 vs. control; Supplementary Fig. S4b), and the respiratory frequency significantly increased (133.2 ± 6.3%, *p* < 0.001 vs. control; Fig. 2f,g), similarly to the changes in the BötC. The duration of both the inspiratory phase and expiratory phase decreased (inspiration, 84.3 ± 2.7%, *p* < 0.001 vs. control; expiration, 80.5 ± 3.7%, *p* < 0.001 vs. control; Fig. 2h,i). The ratio of inspiratory phase duration to respiration showed a small increase (ratio of inspiration in control, 0.23 ± 0.01, with HA, 0.24 ± 0.01, 108.1 ± 3.9%, *p* = 0.043 vs. control; Fig. 2j,k).

### Effects of H_2_S synthesis inhibition at the rVRG

**Figure 3:**
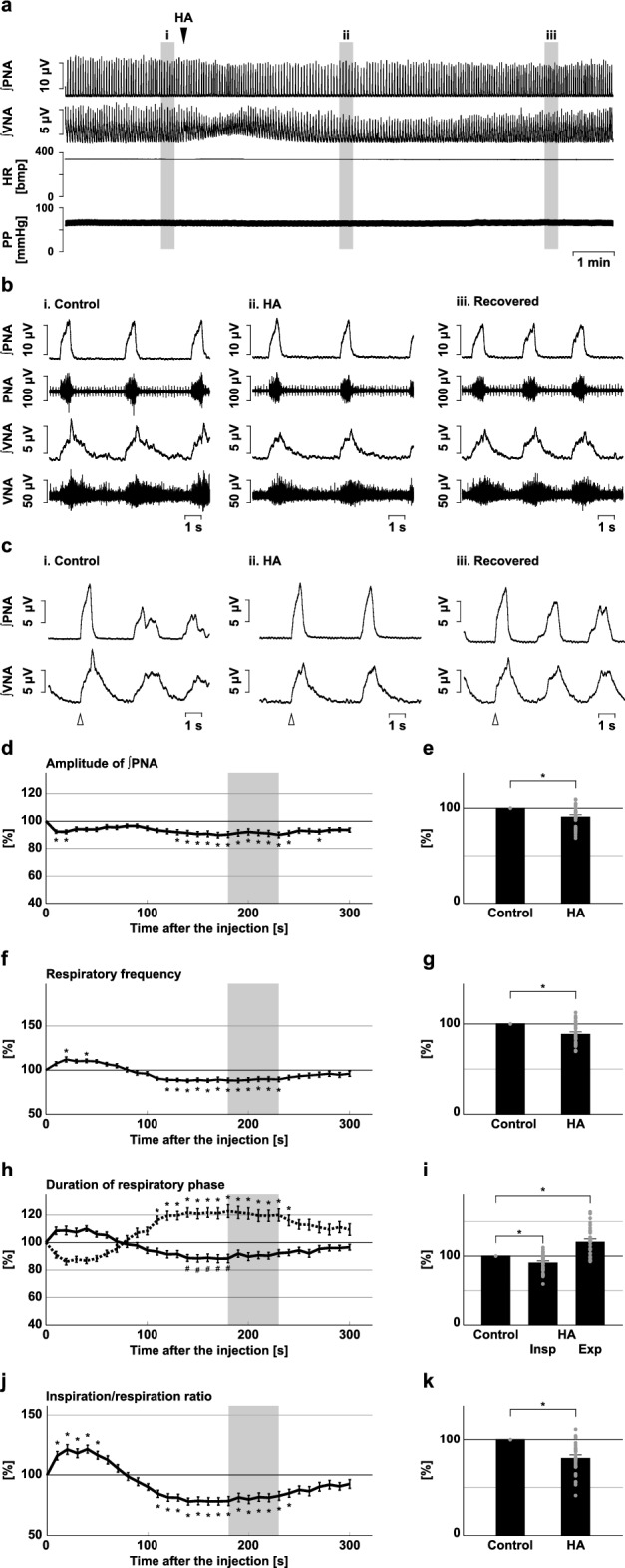
Effects of H_2_S synthesis inhibition at the rVRG on respiration. (**a**) Overall effects of H_2_S synthesis inhibition produced by HA microinjection at the rVRG on the integrated activities of the phrenic nerve (∫PNA) and vagus nerve (∫VNA), heart rate (HR), and perfusion pressure (PP). The triangle indicates the time of drug microinjection. (**b**) Raw waveforms of activities of the phrenic nerve (PNA) and vagus nerve (VNA), ∫PNA, and ∫VNA in the control (i), after HA microinjection (ii), and after recovery (iii). Each waveform was extracted from (i)–(iii) in (**a**). (**c**), Phrenic nerve burst-triggered averages of the ∫PNA and ∫VNA in the control (i), after HA microinjection (ii), and after recovery (iii). Each waveform was obtained by averaging the ∫PNA and ∫VNA during (i)–(iii) in (**a**). The triangle indicates the time of the trigger. (**d**), (**f**), (**h**), (**j**) Temporal changes in the amplitude of ∫PNA (**d**), the respiratory frequency (**f**), the duration of the inspiratory phase (**h**, solid line) and expiratory phase (**h**, dotted line), and the ratio of inspiration to respiration (**j**) (n = 28). (**e**), (**g**), (**i**), (**k**) The change rates of the amplitude of ∫PNA (**e**), the respiratory frequency (**g**), the duration of the inspiratory and expiratory phases (**i**), and the ratio of inspiration to respiration (**k**) after HA microinjection. The change rates were average in the gray area in (**d**), (**f**), (**h**), and (**j**). The asterisks indicate *p* < 0.05 as compared with the control (one sample *t*-test in (**e**), (**g**), (**k**), Dunnett test in (**d**), (**f**), (**h**), (**i**), (**j**)). Results are expressed as means ± SEMs.

HA was also injected into the rVRG (Fig. 3). After microinjection of HA into the rVRG, similarly to the BötC and preBötC, the amplitude of phrenic nerve activity significantly decreased (91.0 ± 2.1%, *p* < 0.001 vs. control; Fig. 3d,e). The amplitude of post-inspiratory vagus nerve activity significantly decreased (92.5 ± 2.9%, *p* = 0.017 vs. control; Supplementary Fig. S4c). In contrast to the BötC and preBötC, the respiratory frequency significantly decreased at the rVRG (89.1 ± 2.4%, *p* < 0.001 vs. control; Fig. 3f,g). The duration of the expiratory phase increased, whilst the duration of the inspiratory phase decreased (expiration, 120.8 ± 4.1%, *p* < 0.001 vs. control; inspiration, 90.5 ± 2.5%, *p* = 0.033 vs. control; Fig. 3h,i). Since the expiratory phase became longer, the ratio of inspiration to respiration decreased (ratio of inspiratory phase in control, 0.20 ± 0.01, with HA, 0.16 ± 0.01, 80.9 ± 3.3%, *p* < 0.001 vs. control; Fig. 3j,k).

### Effects of blockade of excitatory synaptic transmission at the BötC and preBötC

Given the roles of H_2_S as a synaptic modulator, H_2_S may regulate respiratory output via modulation of excitatory and/or inhibitory synaptic transmissions. To confirm this possibility, we examined the effects of blockade of these synaptic transmissions on respiratory output and compared them with the effects of inhibition of H_2_S synthesis.

**Figure 4:**
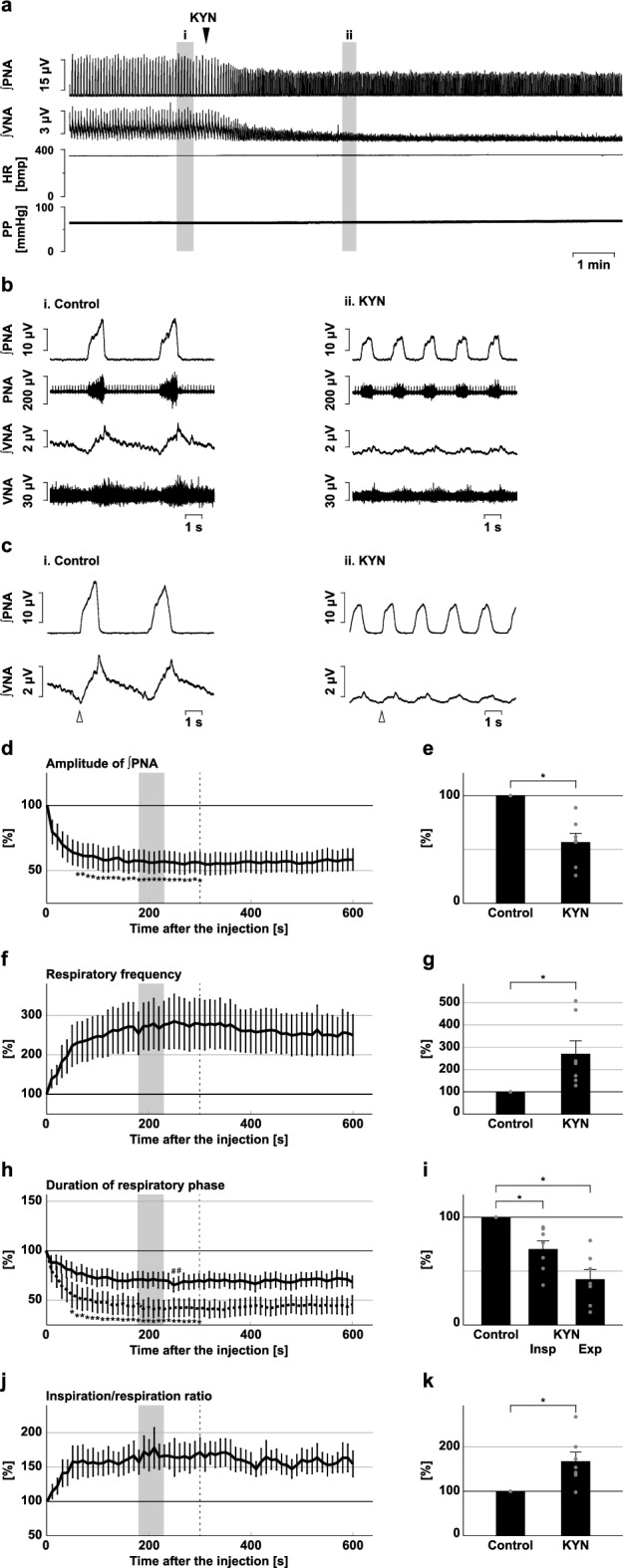
Effects of blockade of excitatory synaptic transmission at the BötC on respiration. (**a**) Overall effects of blockade of excitatory synaptic transmission produced by kynurenic acid (KYN) microinjection at the BötC on the integrated activities of the phrenic nerve (∫PNA) and vagus nerve (∫VNA), heart rate (HR), and perfusion pressure (PP). The triangle indicates the time of drug microinjection. (**b**) Raw waveforms of activities of the phrenic nerve (PNA) and vagus nerve (VNA), ∫PNA, and ∫VNA in the control (i), after KYN microinjection (ii), and after recovery (iii). Each waveform was extracted from (i)–(iii) in (**a**). (**c**), Phrenic nerve burst-triggered averages of the ∫PNA and ∫VNA in the control (i), after KYN microinjection (ii), and after recovery (iii). Each waveform was obtained by averaging the ∫PNA and ∫VNA during (i)–(iii) in (**a**). The triangle indicates the time of the trigger. (**d**), (**f**), (**h**), (**j**) Temporal changes in the amplitude of ∫PNA (**d**), the respiratory frequency (**f**), the duration of the inspiratory phase (**h**, solid line) and expiratory phase (**h**, dotted line), and the ratio of inspiration to respiration (**j**) (n = 7). (**e**), (**g**), (**i**), (**k**) The change rates of the amplitude of ∫PNA (**e**), the respiratory frequency (**g**), the duration of the inspiratory and expiratory phases (**i**), and the ratio of inspiration to respiration (**k**) after KYN microinjection. The change rates were average in the gray areas in (**d**), (**f**), (**h**), and (**j**). The asterisks indicate *p* < 0.05 as compared with the control (one sample *t*-test in (**e**), (**g**), (**k**), Dunnett test in (**d**), (**f**), (**h**), (**i**), (**j**)). The analysis time window in (**d**), (**f**), (**h**), (**j**) was 10–300 s, indicated by the dotted line. Results are expressed as means ± SEMs.

**Figure 5:**
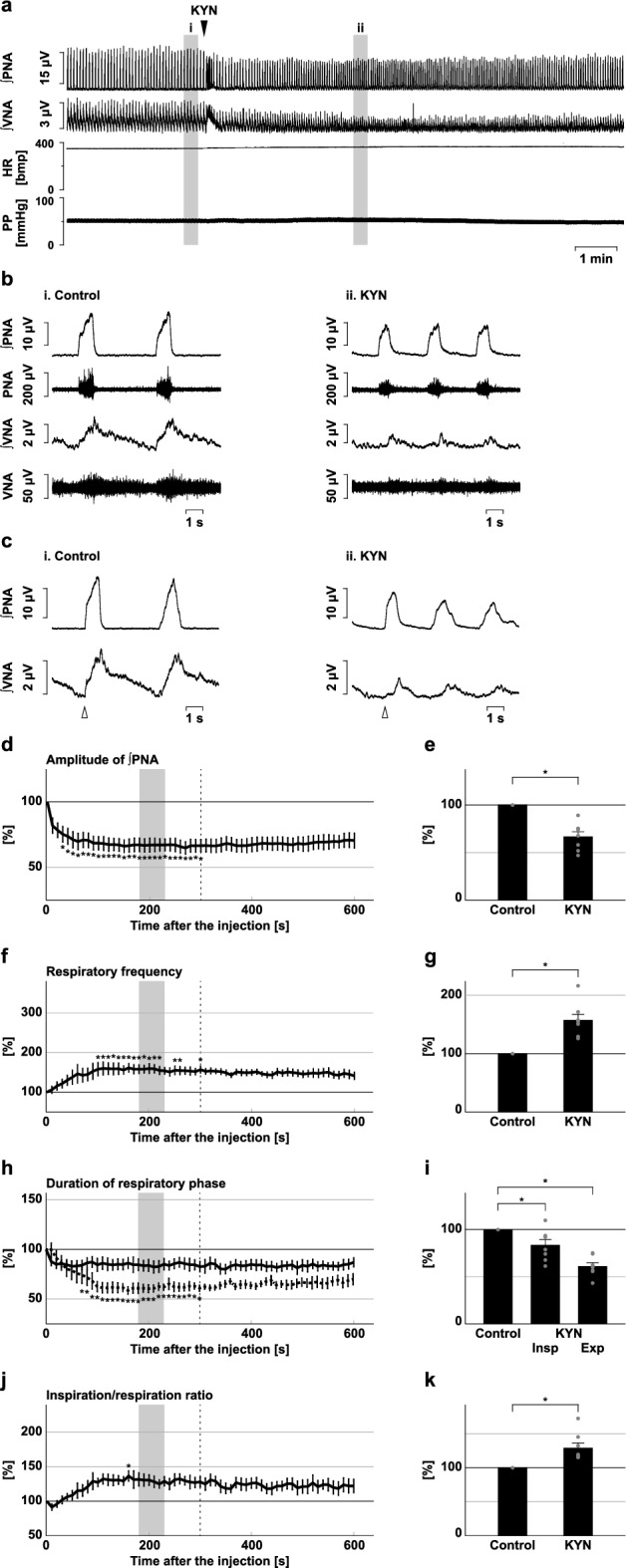
Effects of blockade of excitatory synaptic transmission at the preBötC on respiration. (**a**) Overall effects of blockade of excitatory synaptic transmission produced by kynurenic acid (KYN) microinjection at the preBötC on the integrated activities of the phrenic nerve (∫PNA) and vagus nerve (∫VNA), heart rate (HR), and perfusion pressure (PP). The triangle indicates the time of drug microinjection. (**b**) Raw waveforms of activities of the phrenic nerve (PNA) and vagus nerve (VNA), ∫PNA, and ∫VNA in the control (i), after KYN microinjection (ii), and after recovery (iii). Each waveform was extracted from (i)–(iii) in (**a**). (**c**), Phrenic nerve burst-triggered averages of the ∫PNA and ∫VNA in the control (i), after KYN microinjection (ii), and after recovery (iii). Each waveform was obtained by averaging the ∫PNA and ∫VNA during (i)–(iii) in (**a**). The triangle indicates the time of the trigger. (**d**), (**f**), (**h**), (**j**) Temporal changes in the amplitude of ∫PNA (**d**), the respiratory frequency (**f**), the duration of the inspiratory (**h**, solid line) and expiratory phases (**h**, dotted line), and the ratio of inspiration to respiration (**j**) (n = 8). (**e**), (**g**), (**i**), (**k**) The change rates of the amplitude of ∫PNA (**e**), the respiratory frequency (**g**), the duration of the inspiratory and expiratory phases (**i**), and the ratio of inspiration to respiration (**k**) after KYN microinjection. The change rates were average in the gray areas in (**d**), (**f**), (**h**), and (**j**). The asterisks indicate *p* < 0.05 as compared with the control (one sample *t*-test in (**e**), (**g**), (**k**), Dunnett test in (**d**), (**f**), (**h**), (**i**), (**j**)). The analysis time window in (**d**), (**f**), (**h**), (**j**) was 10–300 s, indicated by the dotted line. Results are expressed as means ± SEMs.

We injected KYN into the BötC (Fig. 4). Blockade of excitatory synaptic transmission at the BötC decreased the phrenic nerve burst amplitude (56.8 ± 8.1%, *p* = 0.002 vs. control; Fig. 4d,e) and the amplitude of post-inspiratory vagus nerve activity (34.4 ± 9.0%, *p* < 0.001 vs. control; Supplementary Fig. S4d), and increased the respiratory frequency (270.6 ± 58.2%, *p* = 0.026 vs. control; Fig. 4f,g). KYN was also applied to the preBötC (Fig. 5). Similarly, after the microinjection of KYN at the preBötC, the amplitude of phrenic nerve activity decreased (67.0 ± 4.9%, *p* < 0.001 vs. control; Fig. 5d,e), the amplitude of post-inspiratory vagus nerve activity decreased (27.5 ± 4.5%, *p* < 0.001 vs. control; Supplementary Fig. S4e), and the respiratory frequency increased (157.5 ± 9.9%. *p* < 0.001 vs. control; Fig. 5f,g). The increased respiratory frequency at the BötC and preBötC resulted from the shortening of both inspiration and expiration (inspiration at the BötC, 70.5 ± 7.7%, *p* = 0.013 vs. control; expiration at the BötC, 42.4 ± 8.9%, *p* < 0.001 vs. control; Fig. 4h,i; inspiration at the preBötC, 83.7 ± 5.7%, *p* = 0.013 vs. control; expiration at the preBötC, 61.2 ± 3.6%, *p* < 0.001 vs. control; Fig. 5h,i). The ratio of inspiration to respiration increased at both the BötC and the preBötC (BötC, 167.6 ± 20.6%, *p* = 0.017 vs. control; Fig. 4j,k; preBötC, 129.1 ± 7.2%, *p* = 0.005 vs. control; Fig. 5j,k). From these results, the trends of the effects of blockade of excitatory transmission at the BötC or preBötC on the respiratory pattern were similar to those of inhibition of H_2_S synthesis in the same regions. Therefore, H_2_S production at the BötC and preBötC contributes to respiratory pattern generation, possibly by sustaining excitatory synaptic transmission.

### Effects of blockade of inhibitory synaptic transmission at the BötC and preBötC

**Figure 6:**
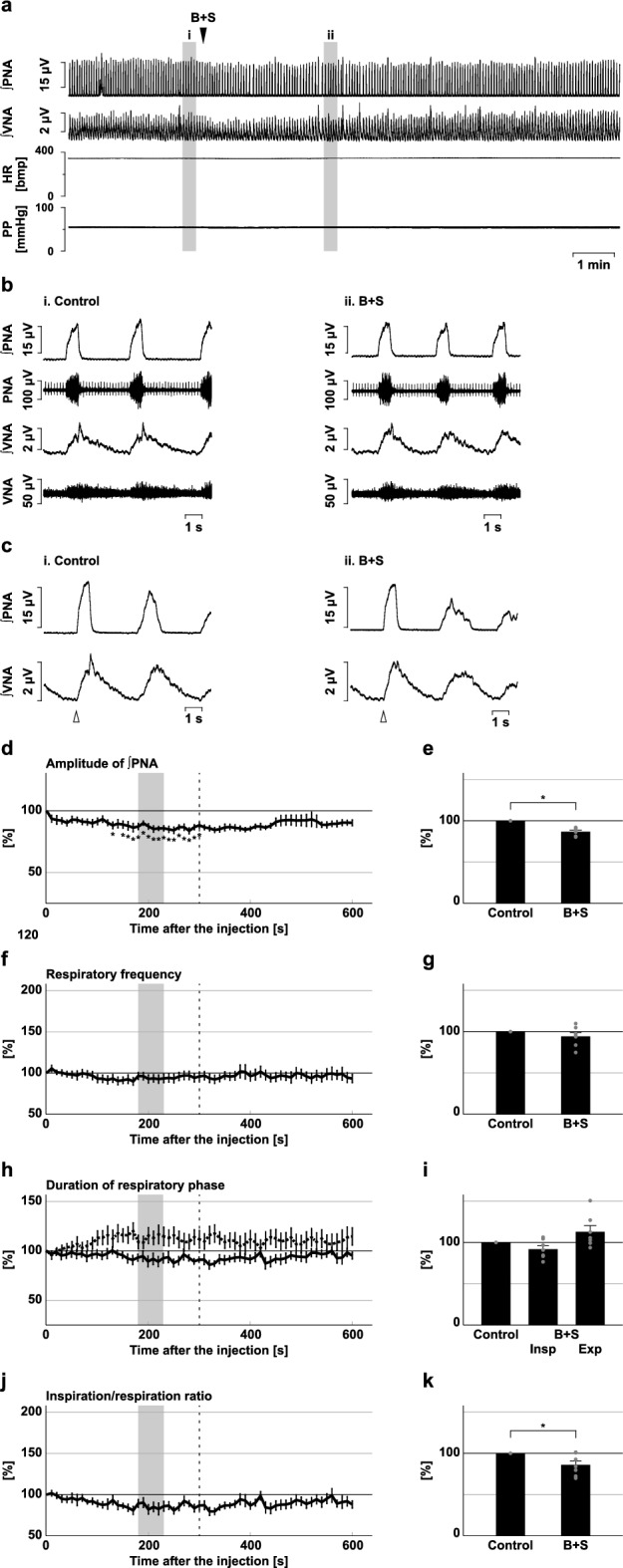
Effects of blockade of inhibitory synaptic transmission at the BötC on respiration. (**a**) Overall effects of blockade of inhibitory synaptic transmission by bicuculline and strychnine (B + S) microinjection at the BötC on the integrated activities of the phrenic nerve (∫PNA) and vagus nerve (∫VNA), heart rate (HR), and perfusion pressure (PP). The triangle indicates the time of drug microinjection. (**b**) Raw waveforms of activities of the phrenic nerve (PNA) and vagus nerve (VNA), ∫PNA, and ∫VNA in the control (i), after B + S microinjection (ii), and after recovery (iii). Each waveform was extracted from (i)–(iii) in (**a**). (**c**), Phrenic nerve burst-triggered averages of the ∫PNA and ∫VNA in the control (i), after B + S microinjection (ii), and after recovery (iii). Each waveform was obtained by averaging the ∫PNA and ∫VNA during (i)–(iii) in (**a**). The triangle indicates the time of the trigger. (**d**), (**f**), (**h**), (**j**) Temporal changes in the amplitude of ∫PNA (**d**), the respiratory frequency (**f**), the duration of the inspiratory phase (**h**, solid line) and expiratory phase (**h**, dotted line), and the ratio of inspiration to respiration (**j**) (n = 7). (**e**), (**g**), (**i**), (**k**) The change rates of the amplitude of ∫PNA (**e**), the respiratory frequency (**g**), the duration of the inspiratory and expiratory phases (**i**), and the ratio of inspiration to respiration (**k**) after B + S microinjection. The change rates were average in the gray areas in (**d**), (**f**), (**h**), and (**j**). The asterisks indicate *p* < 0.05 as compared with the control (one sample *t*-test in (**e**), (**g**), (**k**), Dunnett test in (**d**), (**f**), (**h**), (**i**), (**j**)). The analysis time window in (**d**), (**f**), (**h**), (**j**) was 10–300 s, indicated by the dotted line. Results are expressed as means ± SEMs.

**Figure 7:**
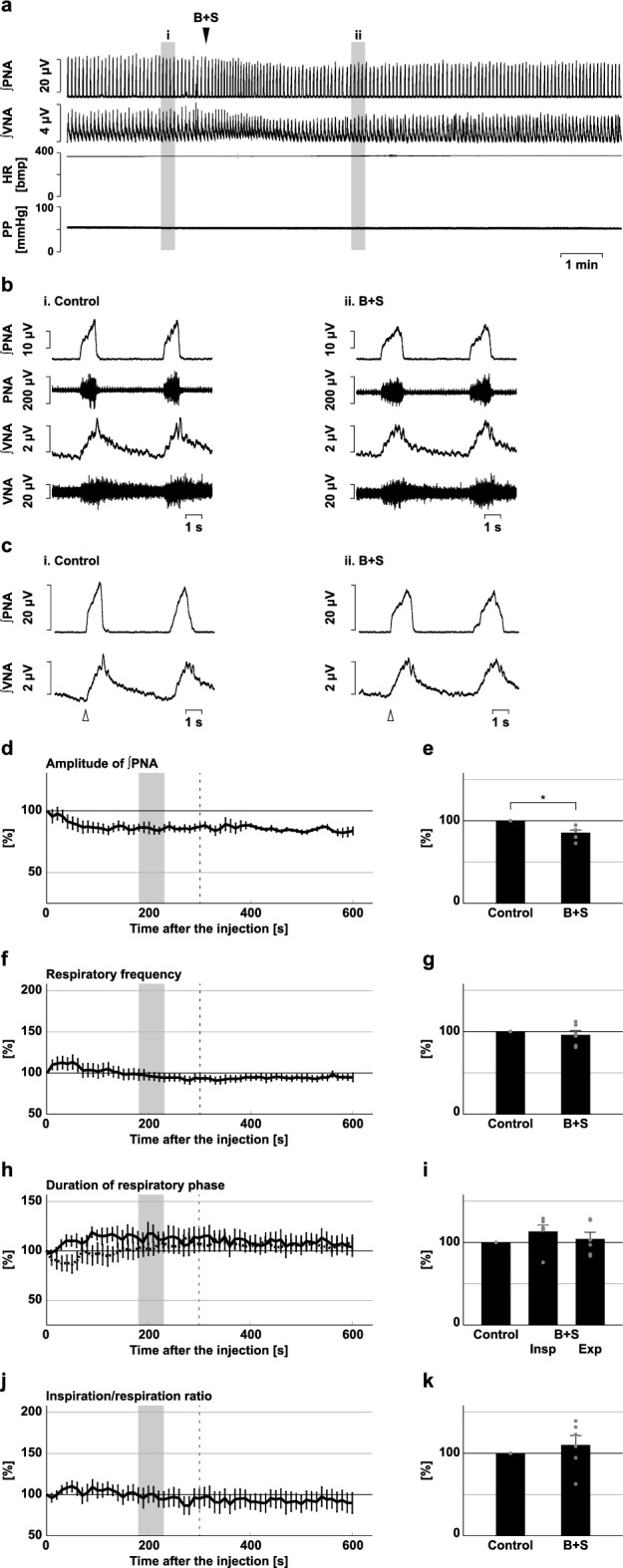
Effects of blockade of inhibitory synaptic transmission at the preBötC on respiration. (**a**) Overall effects of blockade of inhibitory synaptic transmission produced by bicuculline and strychnine (B + S) microinjection at the preBötC on the integrated activities of the phrenic nerve (∫PNA) and vagus nerve (∫VNA), heart rate (HR), and perfusion pressure (PP). The triangle indicates the time of drug microinjection. (**b**) Raw waveforms of activities of the phrenic nerve (PNA) and vagus nerve (VNA), ∫PNA, and ∫VNA in the control (i), after B + S microinjection (ii), and after recovery (iii). Each waveform was extracted from (i)–(iii) in (**a**). (**c**), Phrenic nerve burst-triggered averages of the ∫PNA and ∫VNA in the control (i), after B + S microinjection (ii), and after recovery (iii). Each waveform was obtained by averaging the ∫PNA and ∫VNA during (i)–(iii) in (**a**). The triangle indicates the time of the trigger. (**d**), (**f**), (**h**), (**j**) Temporal changes in the amplitude of ∫PNA (**d**), the respiratory frequency (**f**), the duration of the inspiratory phase (**h**, solid line) and expiratory phase (**h**, dotted line), and the ratio of inspiration to respiration (**j**) (n = 6). (**e**), (**g**), (**i**), (**k**) The change rates of the amplitude of ∫PNA (**e**), the respiratory frequency (**g**), the duration of the inspiratory and expiratory phases (**i**), and the ratio of inspiration to respiration (**k**) after B + S microinjection. The change rates were average in the gray areas in (**d**), (**f**), (**h**), and (**j**). The asterisks indicate *p* < 0.05 as compared with the control (one sample *t*-test in (**e**), (**g**), (**k**), Dunnett test in (**d**), (**f**), (**h**), (**i**), (**j**)). The analysis time window in (**d**), (**f**), (**h**), (**j**) was 10–300 s, indicated by the dotted line. Results are expressed as means ± SEMs.

At both the BötC and at the preBötC, the effect of blockade of inhibitory synaptic transmission by microinjection of bicuculline and strychnine (B + S) on the respiratory pattern was small (Figs. 6 and 7). No significant change in the respiratory frequency occurred after the microinjection of B + S into the BötC or preBötC (BötC, 94.1 ± 4.5%, *p* = 0.242 vs. control; Fig. 6f,g; preBötC, 96.1 ± 5.2%, *p* = 0.482 vs. control; Fig. 7f,g). The duration of each respiratory phase was stable even after B + S microinjection, although the inspiratory ratio to respiration decreased after microinjection of B + S into the BötC (inspiratory duration at the BötC, 91.8 ± 4.2%, *p* = 0.412 vs. control; expiratory duration at the BötC, 112.9 ± 7.5%, *p* = 0.143 vs. control; Fig. 6h,i; inspiratory ratio at the BötC, 85.8 ± 4.8%, *p* = 0.026 vs. control; Fig. 6j,k; inspiratory duration at the preBötC, 113.2 ± 7.8%, *p* = 0.278 vs. control; expiratory duration at the preBötC, 104.2 ± 8.0%, *p* = 0.857 vs. control; Fig. 7h,i; inspiratory ratio at the preBötC, 109.9 ± 11.5%, *p* = 0.427 vs. control; Fig. 7j,k). The amplitude of phrenic nerve activity decreased at both the preBötC and the BötC (BötC, 86.8 ± 1.7%, *p* < 0.001 vs. control; Fig. 6d,e; preBötC, 85.5 ± 3.2%, *p* = 0.006 vs. control; Fig. 7d,e) but the amplitude of post-inspiratory vagus nerve activity did not change (BötC, 91.7 ± 4.1%, *p* = 0.093 vs. control; Supplementary Fig. S4g; preBötC, 97.7 ± 9.6%, *p* = 0.819 vs. control; Supplementary Fig. S4h). Altogether, these results indicate that inhibitory synaptic transmissions at the BötC and preBötC make little contribution to respiratory pattern generation under normal conditions with H_2_S production except for the amplitude of inspiratory burst activity. Therefore, the effects of HA on the respiratory pattern may not be mediated by modulation of inhibitory transmission at the preBötC or BötC.

### Effects of H_2_S synthesis inhibition under blockade of excitatory synaptic transmission

**Figure 8:**
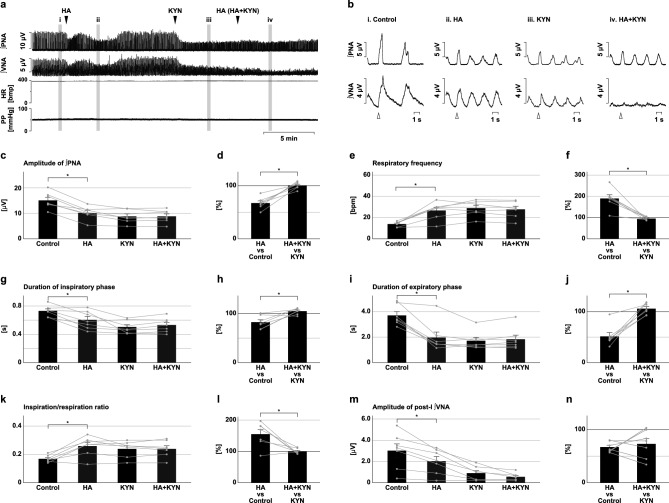
Effects of H_2_S synthesis inhibition in the presence of a blocker of excitatory synaptic transmission at the preBötC. (**a**) Overall effects of H_2_S synthesis inhibition produced by HA microinjection with prior microinjection of KYN at the preBötC on the integrated activities of the phrenic nerve (∫PNA) and vagus nerve (∫VNA), heart rate (HR), and perfusion pressure (PP). The triangles indicate the time of drug microinjections. (**b**), Phrenic nerve burst-triggered averages of ∫PNA and ∫VNA in the control (i), after HA microinjection (ii), after KYN microinjection (iii), and after HA microinjection in the presence of KYN (iv). Each waveform was obtained by averaging the ∫PNA and ∫VNA during (i)–(iv) in (**a**). The triangles indicate the time of the triggers. (**c**), (**e**), (**g**), (**i**), (**k**), (**m**) The amplitude of ∫PNA (**c**), the respiratory frequency (**e**), the duration of the inspiratory phase (**g**) and expiratory phase (**i**), the ratio of inspiration to respiration (**k**), and the amplitude of post-inspiratory ∫VNA (**m**) in the control, after HA microinjection (HA), after microinjection of KYN (KYN), and after HA microinjection in the presence of KYN (HA + KYN, n = 7). (**d**), (**f**), (**h**), (**j**), (**l**), (**n**) The change rates in the amplitude of ∫PNA (**d**), the respiratory frequency (**f**), the duration of the inspiratory phase (**h**) and expiratory phase (**j**), the ratio of inspiration to respiration (**l**), and the amplitude of post-inspiratory ∫PNA (**n**) after HA microinjection in the absence of KYN (HA vs. control) and in the presence of KYN (HA + KYN vs. KYN) to these before HA microinjection. The asterisks indicate *p* < 0.05 as compared between HA vs. control and HA + KYN vs. KYN (paired *t*-test). Results are expressed as means ± SEMs.

**Figure 9:**
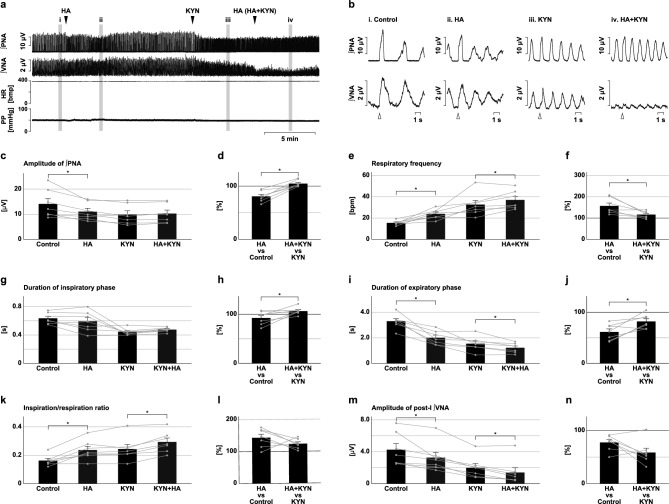
Effects of H_2_S synthesis inhibition in the presence of a blocker of excitatory synaptic transmission at the BötC. (**a**) Overall effects of H_2_S synthesis inhibition produced by HA microinjection with prior microinjection of KYN at the BötC on the integrated activities of the phrenic nerve (∫PNA) and vagus nerve (∫VNA), heart rate (HR), and perfusion pressure (PP). The triangles indicate the time of drug microinjections. (**b**) Phrenic nerve burst-triggered averages of ∫PNA and ∫VNA in the control (i), after HA microinjection (ii), after KYN microinjection (iii), and after HA microinjection in the presence of KYN (iv). Each waveform was obtained by averaging the ∫PNA and ∫VNA during (i)–(iv) in (**a**). The triangles indicate the time of the triggers. (**c**), (**e**), (**g**), (**i**), (**k**), (**m**) The amplitude of ∫PNA (**c**), the respiratory frequency (**e**), the duration of the inspiratory phase (**g**) and expiratory phase (**i**), the ratio of inspiration to respiration (**k**), and the amplitude of post-inspiratory ∫VNA (**m**) in the control, after HA microinjection (HA), after microinjection of KYN (KYN), and after HA microinjection in the presence of KYN (HA + KYN, n = 7). (**d**), (**f**), (**h**), (**j**), (**l**), (**n**) The change rates of the amplitude of ∫PNA (**d**), the respiratory frequency (**f**), the duration of the inspiratory phase (**h**) and expiratory phase (**j**), the ratio of inspiration to respiration (**l**), and the amplitude of post-inspiratory ∫VNA (**n**) after HA microinjection in the absence of KYN (HA vs. control) and in the presence of KYN (HA + KYN vs. KYN) to these before HA microinjections. The asterisks indicate *p* < 0.05 as compared between HA vs. control and HA + KYN vs. KYN (paired *t*-test). Results are expressed as means ± SEMs.

To evaluate the contribution of excitatory synaptic transmission on the changes in the respiratory pattern effected by inhibition of H_2_S synthesis, synthesis of H_2_S was inhibited under the condition of blockade of excitatory synaptic transmission at the preBötC (Fig. 8) and BötC (Fig. 9). At the preBötC, all the effects on respiratory frequency, the duration of each respiratory phase, and the amplitude of the phrenic nerve activity observed after the microinjection of HA were abolished by the prior administration of KYN (amplitude, 100.6 ± 2.4%, *p* = 0.811 vs. KYN; Fig. 8c,d; frequency, 95.6 ± 2.9%, *p* = 0.177 vs. KYN; Fig. 8e,f; inspiratory duration, 104.9 ± 2.3%, *p* = 0.078 vs. KYN; Fig. 8g,h; expiratory duration, 105.8 ± 4.1%, *p* = 0.212 vs. KYN; Fig. 8i,j). Pretreatment of KYN did not change the HA application-induced decrease in the amplitude of post-inspiratory vagus nerve activity (HA vs. control, 67.1 ± 3.6%, HA + KYN vs. KYN, 73.0 ± 9.9%, *p* = 0.588; Fig. 8m,n). At the BötC, the prior microinjection of KYN abolished the effect of HA on the amplitude of phrenic nerve activity (104.7 ± 2.5%, *p* = 0.106 vs. KYN; Fig. 9c,d). Pretreatment of KYN significantly suppressed the HA application-induced increase in respiratory frequency (HA vs. control, 156.9 ± 13.8%, HA + KYN vs. KYN, 116.4 ± 5.9%, *p* = 0.018; Fig. 9e,f) and the decrease in expiratory duration (HA vs. control, 61.5 ± 6.0%, HA + KYN vs. KYN, 82.1 ± 5.2%, *p* = 0.017; Fig. 9i,j) but did not change the decrease in the amplitude of post-inspiratory vagus nerve activity (HA vs. control, 77.5 ± 5.6%, HA + KYN vs. KYN, 58.5 ± 8.2%, *p* = 0.087; Fig. 9m,n). These results indicate that the effects of H_2_S synthesis inhibition at the preBötC and BötC on the respiratory pattern and inspiratory burst amplitude were totally or partially mediated by attenuation of excitatory synaptic transmission.

### Effects of blockade of synaptic transmission at the rVRG

**Figure 10:**
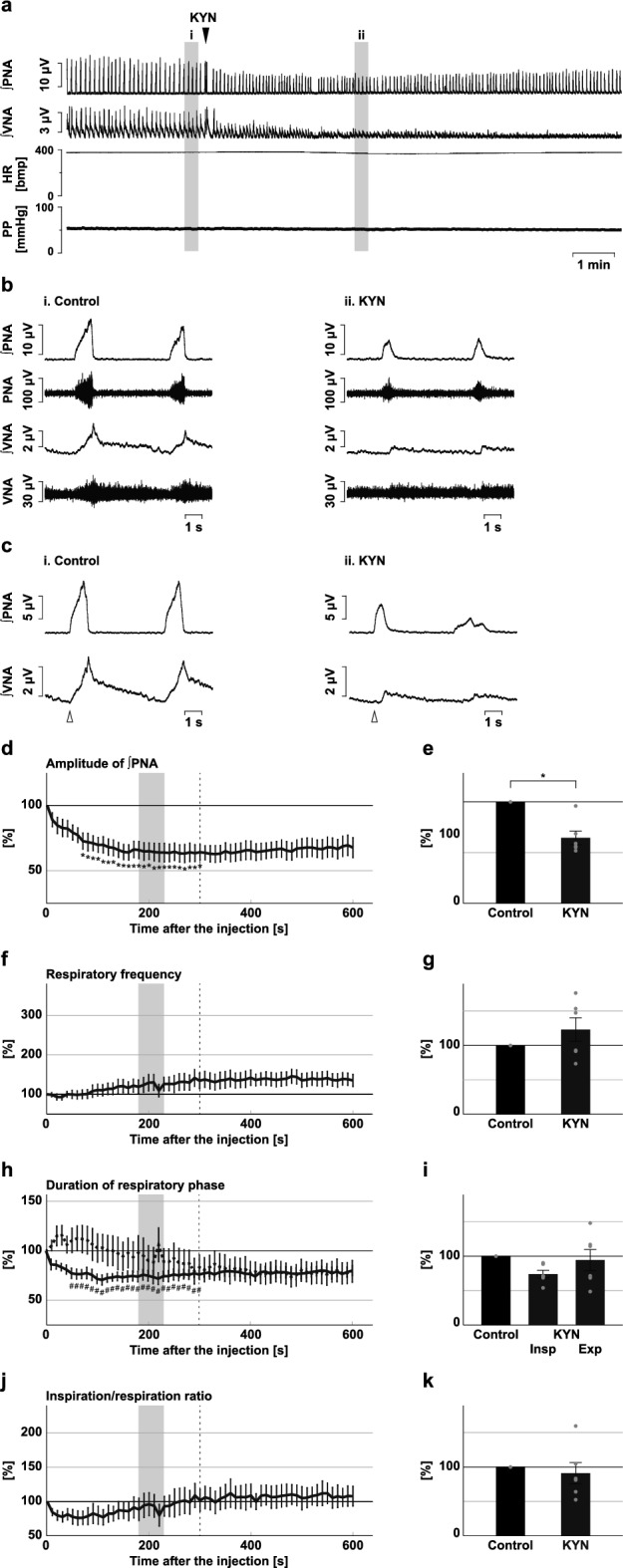
Effects of blockade of excitatory synaptic transmission at the rVRG on respiration. (**a**) Overall effects of blockade of excitatory synaptic transmission produced by kynurenic acid (KYN) microinjection at the rVRG on the integrated activities of the phrenic nerve (∫PNA) and vagus nerve (∫VNA), heart rate (HR), and perfusion pressure (PP). The triangle indicates the time of drug microinjection. (**b**) Raw waveforms of activities of the phrenic nerve (PNA) and vagus nerve (VNA), ∫PNA, and ∫VNA in the control (i), after KYN microinjection (ii), and after recovery (iii). Each waveform was extracted from (i)–(iii) in (**a**). (**c**), Phrenic nerve burst-triggered averages of the ∫PNA and ∫VNA in the control (i), after KYN microinjection (ii), and after recovery (iii). Each waveform was obtained by averaging the ∫PNA and ∫VNA during (i)–(iii) in (**a**). The triangle indicates the time of the trigger. (**d**), (**f**), (**h**), (**j**) Temporal changes in the amplitude of ∫PNA (**d**), the respiratory frequency (**f**), the duration of the inspiratory phase (**h**, solid line) and expiratory phase (**h**, dotted line), and the ratio of inspiration to respiration (**j**) (n = 6). (**e**), (**g**), (**i**), (**k**) The change rates of ∫PNA (**e**), the respiratory frequency (**g**), the duration of the inspiratory and expiratory phases (**i**), and the ratio of inspiration to respiration (**k**) after KYN microinjection. The asterisks indicate *p* < 0.05 as compared with the control (one sample *t*-test in (**e**), (**g**), (**k**), Dunnett test in (**d**), (**f**), (**h**), (**i**), (**j**)). The analysis time window in (**d**), (**f**), (**h**), (**j**) was 10–300 s, indicated by the dotted line. Results are expressed as means ± SEMs.

To confirm the functional role of excitatory transmission at the rVRG, KYN microinjection was applied to the rVRG (Fig. 10). At the rVRG, KYN microinjection decreased the amplitudes of phrenic nerve activity (64.6 ± 6.7%, *p* = 0.003 vs. control; Fig. 10d,e) and post-inspiratory vagus activity (34.9 ± 10.8%, *p* = 0.002 vs. control; Supplementary Fig. S4f), the same as it had at the BötC and preBötC. However, no significant change in respiratory frequency, duration of each respiratory phase, or ratio of inspiration to respiration occurred even after KYN microinjection into the rVRG (frequency, 123.0 ± 17.1%, *p* = 0.238 vs. control; Fig. 10f,g; inspiratory duration, 73.9 ± 5.5%, *p* = 0.121 vs. control; expiratory duration, 94.3 ± 15.4%, *p* = 0.878 vs. control; Fig. 10h,i; inspiratory ratio 90.9 ± 15.6%, *p* = 0.585 vs. control; Fig. 10j,k). These results indicate that excitatory synaptic transmission at the rVRG potentiates the inspiratory burst amplitude but contributes little to respiratory rhythm and pattern generation. Therefore, the attenuation of the inspiratory burst amplitude after inhibition of H_2_S at the rVRG can be explained by attenuation of excitatory transmission, whilst the effects on the respiratory pattern are not likely caused by modulation of excitatory transmission.

**Figure 11:**
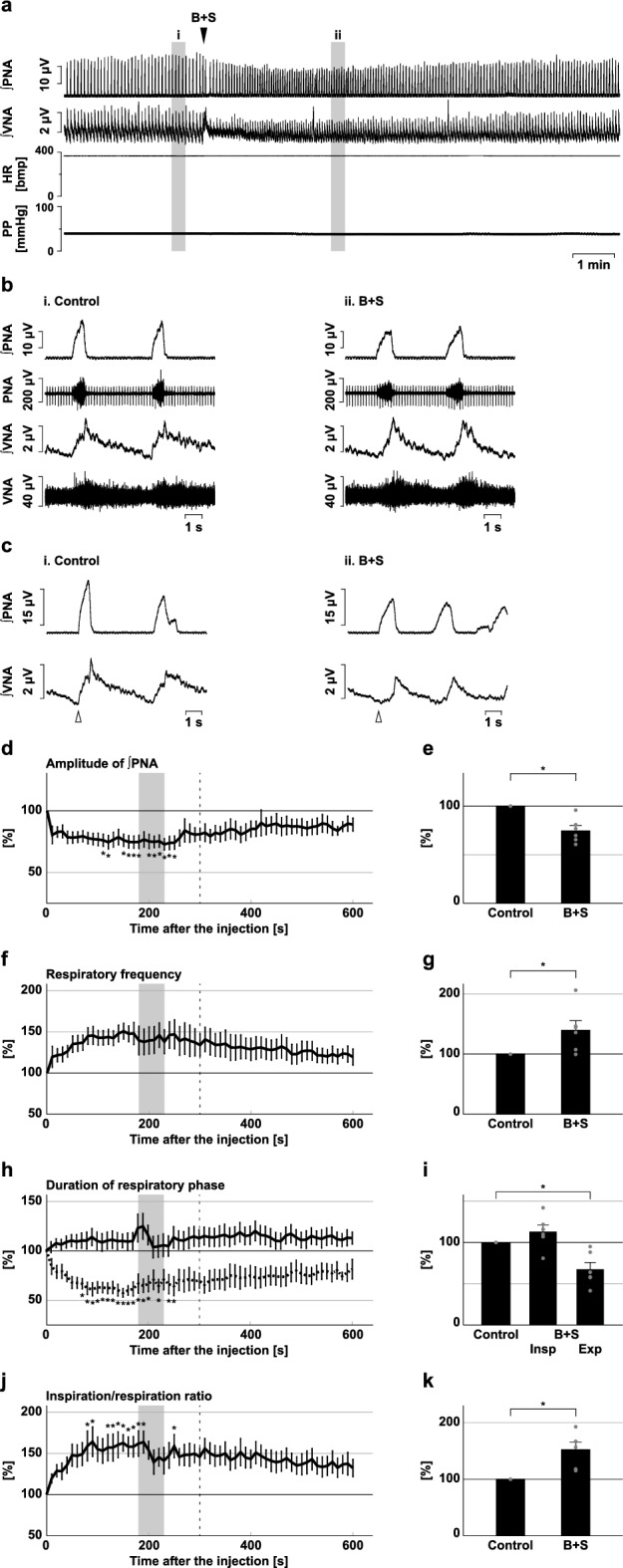
Effects of blockade of inhibitory synaptic transmission at the rVRG on respiration. (**a**) Overall effects of blockade of inhibitory synaptic transmission produced by bicuculline and strychnine (B + S) microinjection at the rVRG on the integrated activities of the phrenic nerve (∫PNA) and vagus nerve (∫VNA), heart rate (HR), and perfusion pressure (PP). The triangle indicates the time of drug microinjection. (**b**) Raw waveforms of activities of the phrenic nerve (PNA) and vagus nerve (VNA), ∫PNA, and ∫VNA in the control (i), after B + S microinjection (ii), and after recovery (iii). Each waveform was extracted from (i)–(iii) in (**a**). (**c**), Phrenic nerve burst-triggered averages of the ∫PNA and ∫VNA in the control (i), after B + S microinjection (ii), and after recovery (iii). Each waveform was obtained by averaging the ∫PNA and ∫VNA during (i)–(iii) in (**a**). The triangle indicates the time of the trigger. (**d**), (**f**), (**h**), (**j**) Temporal changes in the amplitude of ∫PNA (**d**), the respiratory frequency (**f**), the duration of the inspiratory phase (**h**, solid line) and expiratory phase (**h**, dotted line), and the ratio of inspiration to respiration (**j**) (n = 6). (**e**), (**g**), (**i**), (**k**) The change rates of the amplitude of ∫PNA (**e**), the respiratory frequency (**g**), the duration of the inspiratory and expiratory phases (**i**), and the ratio of inspiration to respiration (**k**) after B + S microinjection. The change rates were average in the gray area in (**d**), (**f**), (**h**), and (**j**). The asterisks indicate *p* < 0.05 as compared with the control (one sample *t*-test in (**e**), (**g**), (**k**), Dunnett test in (**d**), (**f**), (**h**), (**i**), (**j**)). The analysis time window in (**d**), (**f**), (**h**), (**j**) was 10–300 s, indicated by the dotted line. Results are expressed as means ± SEMs.

Next, to confirm the functional role of inhibitory transmission at the rVRG, B + S microinjection was also administered to the rVRG (Fig. 11). After blockade of inhibitory synaptic transmission by B + S microinjection into the rVRG, both the amplitude of the inspiratory burst and the respiratory pattern changed. The amplitude of phrenic nerve activity decreased (74.9 ± 5.2%, *p* = 0.005 vs. control; Fig. 11d,e) but the amplitude of post-inspiratory vagus nerve activity did not change (79.0 ± 9.6%, *p* = 0.080 vs. control; Supplementary Fig. S4i). The respiratory frequency increased (140.1 ± 15.5%, *p* = 0.049; Fig. 11f,g). The increased respiratory frequency mainly resulted from the shortening of the expiratory phase duration (67.4 ± 8.3%, *p* = 0.007 vs. control; Fig. 11h,i). Owing to the significant effects on the expiratory phase rather than on the inspiratory phase, the ratio of inspiration to respiration increased (153.0 ± 12.4%, *p* = 0.008 vs. control; Fig. 9j,k). The response directions of blockade of inhibitory synaptic transmission on the respiratory pattern, especially on respiratory frequency, duration of the expiratory phase, and ratio of inspiration to respiration, were opposite to those of inhibition of H_2_S synthesis.

Altogether, these results suggest that H_2_S at the rVRG contributes to the generation of inspiration by facilitating excitatory synaptic transmission, whilst it contributes to respiratory pattern generation by attenuating inhibitory transmission.

## Discussion

Here we investigated the functional roles of H_2_S synthesized at each subregion of the medullary respiratory network in respiratory pattern generation in an in situ arterially perfused preparation of rats. Microinjection of a CBS inhibitor into the preBötC and BötC attenuated the inspiratory burst and increased the respiratory frequency with shorter inspiration and expiration, respectively. These effects were similar to those obtained by blockade of excitatory synaptic transmission at the preBötC or BötC, and the effects of the CBS inhibitor were abolished or attenuated in the presence of a blocker of excitatory synaptic transmission (Table 1). Therefore, H_2_S synthesized at the preBötC and BötC functions to limit respiratory frequency by sustaining the duration of the inspiratory and expiratory phases, respectively, and by maintaining the power of inspiration, possibly by facilitating excitatory transmission. On the other hand, at the rVRG, microinjection of the CBS inhibitor attenuated the amplitude of the inspiratory burst and decreased the respiratory frequency with a longer interinspiration interval. Whilst blockade of excitatory synaptic transmission at the rVRG attenuated only the amplitude of the inspiratory burst, blockade of inhibitory synaptic transmission at the rVRG increased the respiratory frequency with a shorter interinspiration interval (Table 1). These results suggest that H_2_S synthesized at the rVRG contributes to the power of inspiration and to respiratory rhythm, possibly by facilitating excitatory synaptic transmission and attenuating inhibitory synaptic transmission, respectively.

**Table 1:** Effects of HA, KYN, and B + S microinjection at the BötC, preBötC, and rVRG on respiration.

	BötC				preBötC				rVRG		
	HA		KYN	B + S	HA		KYN	B + S	HA	KYN	B + S
	Without KYN	With KYN			Without KYN	With KYN					
Respiratory frequency	↑	Suppressed	↑	–	↑	Suppressed	↑	–	↓	–	↑
Duration of respiratory phases	Exp↓	Suppressed	Exp↓	–	Insp↓ Exp↓	Suppressed	Insp↓ Exp↓	–	Exp↑	–	Exp↓
Inspiration/respiration ratio	↑	Suppressed	↑	–	–	Suppressed	–	–	↓	–	↑
Amplitude of inspiratory PNA	↓	Suppressed	↓	↓	↓	Suppressed	↓	↓	↓	↓	↓
Amplitude of post-inspiratory VNA	↓	↓	↓	–	↓	↓	↓	–	↓	↓	–

↑, increase; ↓, decrease; –, no change. “Suppressed” means that effects by HA were suppressed in the presence of KYN.

The medullary respiratory network is composed of various types of neurons according to respiratory phase-related activity^24^. The spatial differences in the distribution of those neurons result in the different roles of each respiratory compartment, the BötC, the preBötC, and the rVRG, in respiratory pattern generation^11,25,26^. Therefore, we hypothesized that the contribution of endogenous H_2_S production to the generation of respiratory output differs according to each respiratory compartment. To test this hypothesis, we locally modulated the synthesis of H_2_S in the BötC, preBötC, and rVRG. In the BötC and preBötC, which are the essential centers of respiratory pattern generation, the lack of endogenous H_2_S production attenuated the power of inspiration and post-inspiration represented by the amplitude of the burst of phrenic and post-inspiratory vagus nerve activity and increased respiratory frequency. Dividing respiration into inspiratory and expiratory phases, we found differences in the effects of H_2_S synthesis inhibition at the BötC and preBötC on each respiratory phase, which might depend on the roles of the BötC and preBötC in respiratory pattern generation.

The present study showed that the lack of endogenous H_2_S production in the BötC led to an inability to sustain expiration. Despite the increase in respiratory frequency, the duration of the inspiratory phase was almost maintained. Thus, the ratio of inspiration to respiration increased. This finding indicates that endogenous H_2_S production in the BötC is crucial in sustaining expiratory phase duration. The BötC is a region for generation of the expiratory phase with abundant expiratory neurons, and this region's excitation prolongs expiratory phase duration^27,28^. H_2_S production modulates synaptic transmission, and it was previously reported that it can regulate neural networks to maintain respiration^7^. Moreover, it has been reported that the sparse neural network at the respiratory network increases respiratory frequency and decreases the inspiratory burst^29^. Thus, we hypothesized that the lack of H_2_S production in the BötC shortened the expiratory phase duration by altering synaptic transmission. To test this hypothesis, firstly, we microinjected a blocker of excitatory or inhibitory synaptic transmission and evaluated the effect on respiratory output. Secondly, we investigated whether respiratory output is changed by an H_2_S synthase inhibitor even after synaptic transmission is blocked. We found that blockade of excitatory synaptic transmission, not of inhibitory transmission, shortened the expiratory phase duration, similarly to the effect obtained by H_2_S synthesis inhibition. Moreover, this change was attenuated in the presence of the blocker of excitatory synaptic transmission. Therefore, the change in expiratory phase duration after inhibition of H_2_S synthesis at the BötC might be mediated by the attenuation of excitatory synaptic transmission. Several studies have reported that endogenous H_2_S production can facilitate synaptic transmission by increasing the release of synaptic transmitters or modulating the receptor function^2,5^. Therefore, although we need further experiments, it is likely that endogenous H_2_S production has facilitatory roles in excitatory synaptic transmission at the medullary respiratory network. In addition, inhibition of H_2_S synthesis in the BötC decreased the inspiratory burst amplitude, indicating that H_2_S production in the BötC contributes to the potentiation of inspiratory power. On the basis of our results showing that blockade of excitatory synaptic transmission decreased inspiratory burst amplitude, the change in the inspiratory burst amplitude by H_2_S synthesis inhibition may also be caused by the attenuation of excitatory synaptic transmission.

At the preBötC, a kernel region for generation of inspiration, the lack of endogenous H_2_S production shortened the duration of the inspiratory phase and attenuated the inspiratory burst amplitude. This finding indicates that endogenous H_2_S production in the preBötC has a role in sustaining the power of inspiration and limiting the respiratory frequency by maintaining the duration of the inspiratory phase. The shorter and weaker inspiration after inhibition of H_2_S synthase possibly results from attenuation of the activities of inspiratory neurons distributed predominantly in this region. On the basis of our results, it is likely caused by attenuation of excitatory synaptic transmission, not by inhibitory transmission. After excitatory synaptic transmission at the preBötC had been blocked, the duration of the inspiratory phase was shortened. Moreover, in the presence of the blocker of excitatory synaptic transmission, inhibition of H_2_S synthase did not affect the duration and power of inspiration. Therefore, these findings suggest that H_2_S production in the preBötC potentiates excitatory synaptic transmission to sustain the duration and power of inspiration. Excitatory synaptic transmission may increase the excitability of inspiratory neurons and accumulate inspiratory power. Surprisingly, inhibition of H_2_S synthase in the preBötC shortened not only the duration of the inspiratory phase but also the duration of the expiratory phase. This finding indicates that H_2_S in the preBötC contributes to sustaining the duration of the expiratory phase as well as of inspiration. Herein, we showed that blockade of excitatory synaptic transmission decreased the duration of the expiratory phase, whereas blockade of inhibitory synaptic transmission did not affect the duration of each respiratory phase. Bilateral blockade of inhibitory synaptic transmission in the larger area of the preBötC or BötC changes the respiratory frequency and pattern^23^. Therefore, our finding indicates that loss of inhibitory synaptic transmission at a limited area of the preBötC does not affect eupneic respiration. In other words, inhibitory transmission contributes less to respiratory pattern generation. This finding is consistent with that of a previous report showing that postsynaptic inhibition within the BötC and preBötC is not essential for the generation of normal respiratory rhythm^20^. Thus, H_2_S production in the preBötC sustains the duration of the expiratory phase possibly by potentiating excitatory synaptic transmission in the preBötC, not by altering inhibitory synaptic input from BötC expiratory neurons. The shorter inspiration may decrease the interinspiration interval by shortening the inactivated period of inspiratory neurons^30^. It might also be because some expiratory neurons are distributed in the preBötC, although the number of expiratory neurons in the preBötC is smaller than that in the BötC^30–32^.

We also evaluated the role of H_2_S synthesized in the rVRG containing bulbospinal inspiratory premotor neurons. When the H_2_S synthase inhibitor was microinjected into the rVGR, the amplitude of the inspiratory burst decreased. This change may be caused by the suppression of inspiratory premotor neurons and their synchronization, similarly to the preBötC or the BötC. Surprisingly, it also changed the respiratory frequency and the balance of inspiration and expiration. Decrease in respiratory frequency was caused by the delay of inspiratory onset with a longer interinspiration interval. This finding indicates that inspiration is less likely to be generated under the lack of H_2_S in the rVRG. Therefore, H_2_S synthesized in the rVRG functions to potentiate the burst power and rhythm of inspiration. When excitatory transmission was blocked in the rVRG, only the amplitude of the inspiratory burst decreased; no changes occurred in the respiratory frequency, duration of each respiratory phase, or ratio of inspiration to respiration. Thus, the changes in the respiratory pattern effected by H_2_S synthesis inhibition at the rVRG were not fully caused by blockade of excitatory transmission. On the other hand, blockade of inhibitory synaptic transmission at the rVRG decreased the amplitude of the inspiratory burst and increased the respiratory frequency with a shorter expiration, resulting in an increase in the ratio of inspiration to respiration. These alterations of the respiratory pattern were almost opposite to those produced by H_2_S synthesis inhibition at the rVRG other than the amplitude of the inspiratory burst. It seems that H_2_S synthesis inhibition at the rVRG potentiates inhibitory synaptic transmission, disrupting the inspiration-expiration balance. From these observations, endogenous H_2_S production at the rVRG potentiates the inspiratory burst power possibly by facilitating excitatory synaptic transmission, whilst it accelerates inspiratory rhythm possibly by attenuating inhibitory synaptic transmission. Although the preBötC and BötC are responsible regions for respiratory rhythm generation based on the respiratory pattern generation network model, some studies have reported that excitation of rVRG neurons can alter respiratory frequency by modulating the duration of the respiratory phase, indicating that the rVRG also contributes to respiratory rhythm generation^17,33^. The rVRG receives inhibitory inputs from BötC expiratory neurons^33,34^. Together with our result showing that administration of GABAergic and glycinergic receptors antagonists into the rVRG alters respiratory frequency and duration of expiration, the inhibitory synaptic inputs into the rVRG may contribute to respiratory rhythm stabilization and these inputs could be modulated by endogenous H_2_S. However, because there is no report regarding the modulatory role of H_2_S in inhibitory synaptic transmission, we need further experiments to confirm whether this is the case.

At the BötC, preBötC, and rVRG, inhibition of endogenous H_2_S production decreased the amplitude of post-inspiratory vagus nerve activity. This indicates that endogenous H_2_S production in the ventral respiratory column has a role in sustaining post-inspiratory power. Interestingly, the decrease in post-inspiratory power induced by H_2_S inhibition at the BötC and preBötC was preserved under the blockade of excitatory transmission by KYN, although the microinjection of KYN also decreased the amplitude of post-inspiratory vagus nerve activity. This suggested that H_2_S production facilitates the glutamatergic and non-glutamatergic excitatory post-inspiratory synaptic transmission at the medullary respiratory network. Recently, one of the brain regions involved in controlling post-inspiration has been identified in the reticular formation of the medulla, the post-inspiratory complex (PiCo)^14,35^. The PiCo neurons consist of glutamatergic and cholinergic neurons and have reciprocal connections between the ventral respiratory column and the PiCo, although the functional roles of these connections remain unclear^36–38^. Therefore, endogenous H_2_S might facilitate glutamatergic and cholinergic post-inspiratory inputs to the ventral respiratory column from the PiCo.

In the present study, we cannot exclude the possibility that endogenous H_2_S production directly excites neurons. However, previous studies focusing on the direct effect of H_2_S on neurons have found that H_2_S can activate the K_ATP_ channel and cause hyperpolarization^4,39–41^. In this study, we found that the effects of increasing the excitability of neurons by blocking the inhibitory synaptic transmission on the respiratory pattern completely differed from those of H_2_S synthesis inhibition. Therefore, it is unlikely that the hyperpolarization via activation of the K_ATP_ channel is the underlying mechanism of respiratory regulation by endogenous H_2_S production at the medullary respiratory network. To confirm whether endogenous H_2_S production directly excites neurons, we need further studies concerning the involvement of the AC-cAMP pathway or other possible mechanisms^3,42^.

H_2_S in the central nervous system is synthesized by cystathionine β-synthase (CBS), which is expressed in the brainstem and spinal cord^43–45^. To modulate the synthesis of H_2_S, we used HA, which is widely used as a CBS inhibitor. It can inhibit CBS by inactivating heme, which is a cofactor of CBS. Previous studies have confirmed that HA dose-dependently suppresses the production of H_2_S^1,46^. Therefore, although we did not measure the concentration of H_2_S in this study, we considered that the local level of H_2_S gradually decreased after the microinjection of HA and that it recovered by diffusion. Although HA can also inhibit cystathionine γ-lyase (CSE), the other H_2_S synthase, it does not affect our conclusion because CSE is mainly expressed in peripheral tissue and less in the central nervous system^1,46^. Additionally, a previous study showed that a CSE-selective inhibitor does not affect the respiratory pattern^7^. Therefore, it seems that the effects of HA microinjection into the medullary respiratory network in this study caused the reduction in H_2_S synthesis mediated by inhibition of CBS, not of CSE.

The amount of H_2_S in the brain is modulated under physiologic conditions, such as hypoxia or hypercapnia. Some studies have reported that H_2_S production levels in various brain areas, such as the NTS and medial raphe, increase under hypercapnic or hypoxic conditions^47–49^, and another study also showed that the H_2_S level in the BötC decreases under hypoxia^9^. In addition, the pharmacologic modulation of H_2_S levels affects ventilatory responses to hypoxic or hypercapnic conditions^8–10^. Therefore, H_2_S changes respiratory output, possibly by modulating the synthesis of H_2_S depending on the brain areas and physiologic conditions. In this study, we revealed that the H_2_S in each subregion of the medullary respiratory network dependently contributes to basal respiratory outputs by potentiating the neural network via facilitation of excitatory synaptic transmission and/or attenuation of inhibitory synaptic transmission. These mechanisms might also be involved in the ventilatory response to hypoxia or hypercapnia or in pathologic respiratory changes depending on the subregion of the medullary respiratory network.

In conclusion, this study revealed that H_2_S synthesized in the medullary respiratory network functions to maintain the duration of inspiration and expiration and the power of the inspiration site dependently. H_2_S synthesized in the preBötC and BötC functions to limit respiratory frequency by sustaining inspiration and expiration, respectively, and maintains the power of inspiration. In contrast, H_2_S synthesized in the rVRG functions to promote respiratory frequency by modulating the interval of inspiration and maintaining the power of inspiration. These functions might result from the facilitation of excitatory synaptic transmission and/or the attenuation of inhibitory synaptic transmission. Thus, H_2_S production in the medullary respiratory network is an endogenous key modulator for respiratory pattern and rhythm generation.

### Supplementary Information

Supplementary Information.

## Data Availability

The data that support the findings of this study are available from the corresponding author upon reasonable request.
