# Supplementary material for: Hydrogen sulfide production in the medullary respiratory center modulates the neural circuit for respiratory pattern and rhythm generations
Source: Sci Rep. 2023 Dec 4;13:20046. doi: 10.1038/s41598-023-47280-9 (PMC10696040; doi:10.1038/s41598-023-47280-9)
Supplement: Supplementary file 1 — Supplementary Information. [file 41598_2023_47280_MOESM1_ESM.pdf]

Supplemental Figures

Title: **Hydrogen sulfide production in the medullary respiratory center modulates the neural circuit for respiratory pattern and rhythm generations**

Authors: Minako Okazaki[1,2], Masayuki Matsumoto[3,4], and Tadachika Koganezawa[1,4*]

Author addresses: 1. Department of Neurophysiology, Division of Biomedical Science, Institute of Medicine, University of Tsukuba, Tsukuba, Ibaraki 305-8575, Japan

2. Doctoral Program in Neuroscience, Graduate School of Comprehensive Human Sciences, University of Tsukuba, Tsukuba, Ibaraki 305-8575, Japan

3. Department of Cognitive and Behavioral Neuroscience, Division of Biomedical Science, Institute of Medicine, University of Tsukuba, Tsukuba, Ibaraki 305-8575, Japan

4. Transborder Medical Research Center, University of Tsukuba, Tsukuba, Ibaraki 305-8575, Japan

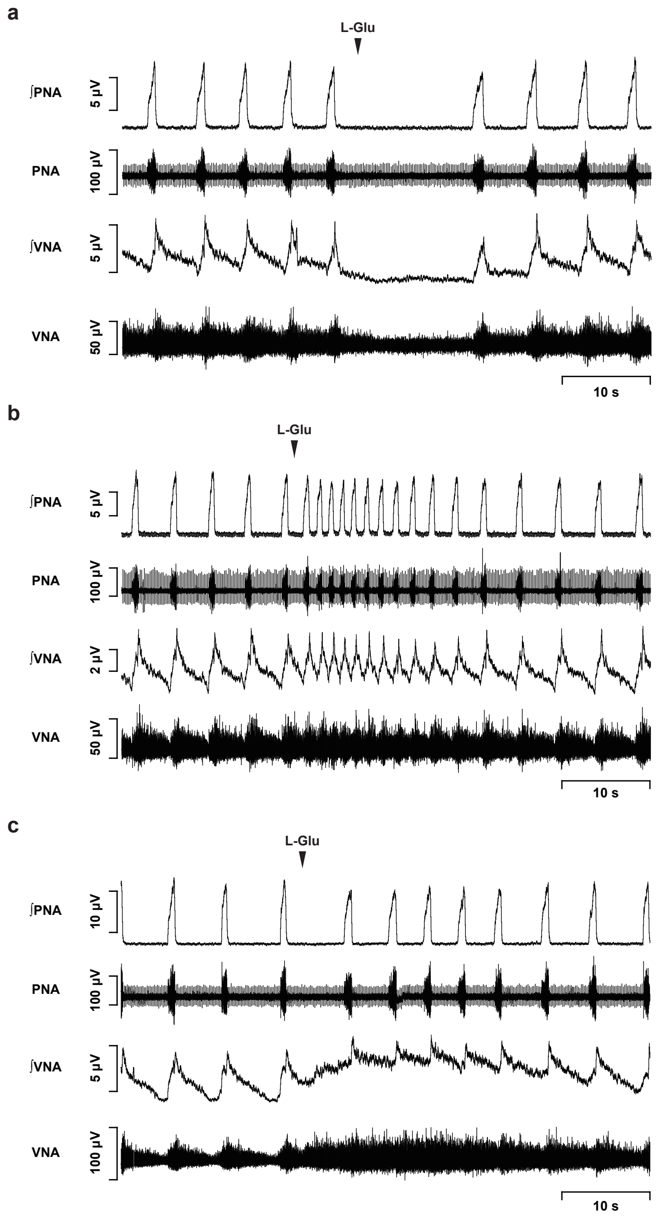

Figure S1.

Respiratory responses to the L-glutamate microinjection at each respiratory center

(a), (b), (c) An example of the effects of L-glutamate microinjection into the BötC (a), preBötC (b), and rVRG (c) on the integrated activities of the phrenic nerve (∫PNA), PNA, integrated activities of the vagus nerve (∫VNA), and VNA. The triangle indicates the time of drug microinjection.

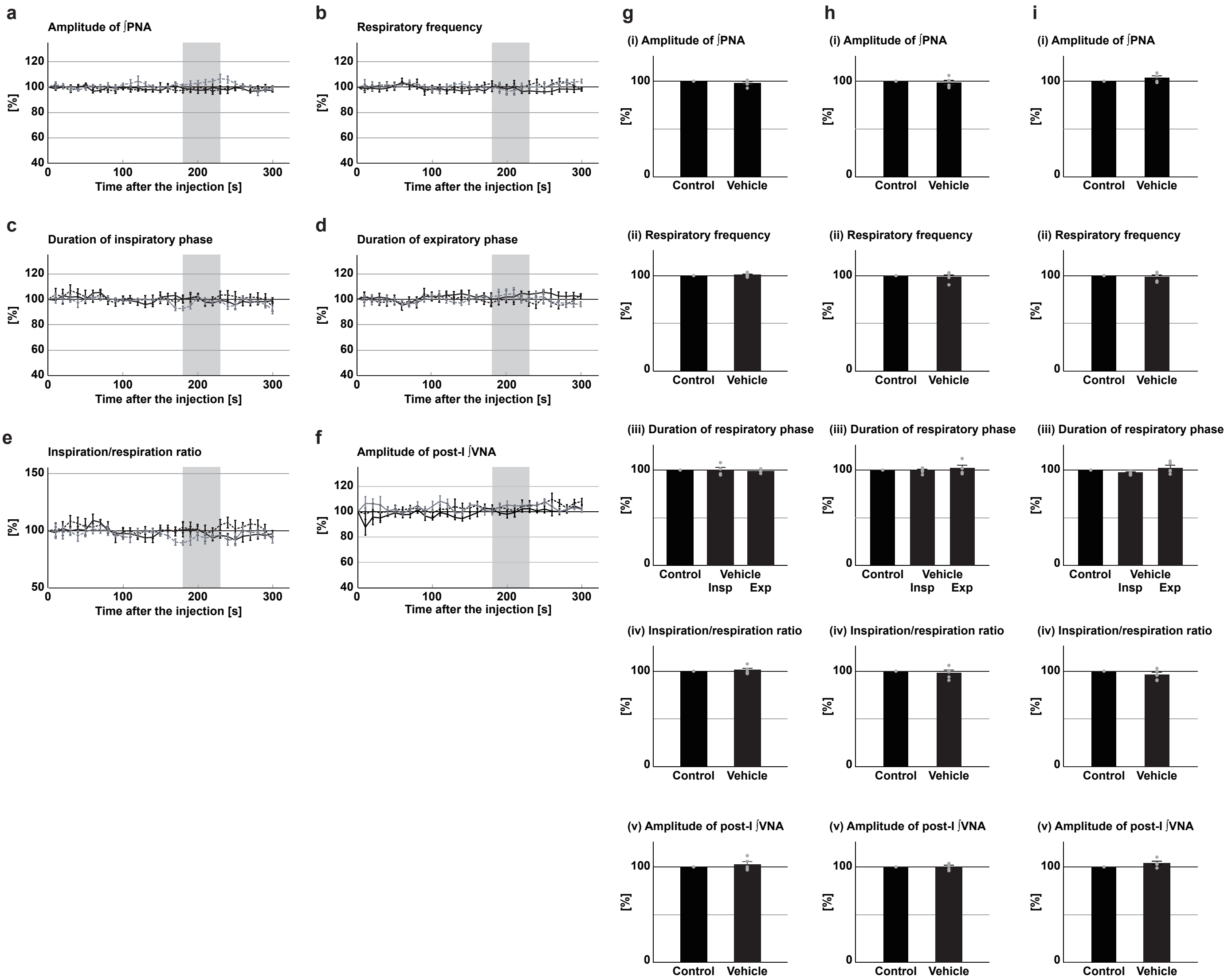

Figure S2.

Effects of vehicle injection at the BötC, preBötC, and rVRG on respiration

(a), (b), (c), (d), (e), (f) Temporal changes in the amplitude of the integrated activities of the phrenic nerve (∫PNA) (a), the respiratory frequency (b), the duration of the inspiratory phase (c) and the expiratory phase (d), the ratio of inspiration to respiration (e), and the amplitude of post-inspiratory ∫VNA (f) after vehicle microinjection at the BötC (black dotted line, n = 5), preBötC (black solid line, n = 5), and rVRG (gray dotted line, n = 5). **(g), (h), (i)** Change rates of the amplitude of ∫PNA (i), the respiratory frequency (ii), the duration of the inspiratory and expiratory phases (iii), the ratio of inspiration to respiration (iv), and the amplitude of post-inspiratory ∫VNA (v) after vehicle microinjection at the BötC (g), preBötC (h), rVRG (i). The change rates were average in the gray areas in (a), (b), (c), (d), (e), and (f). Results are expressed as means ± SEMs.

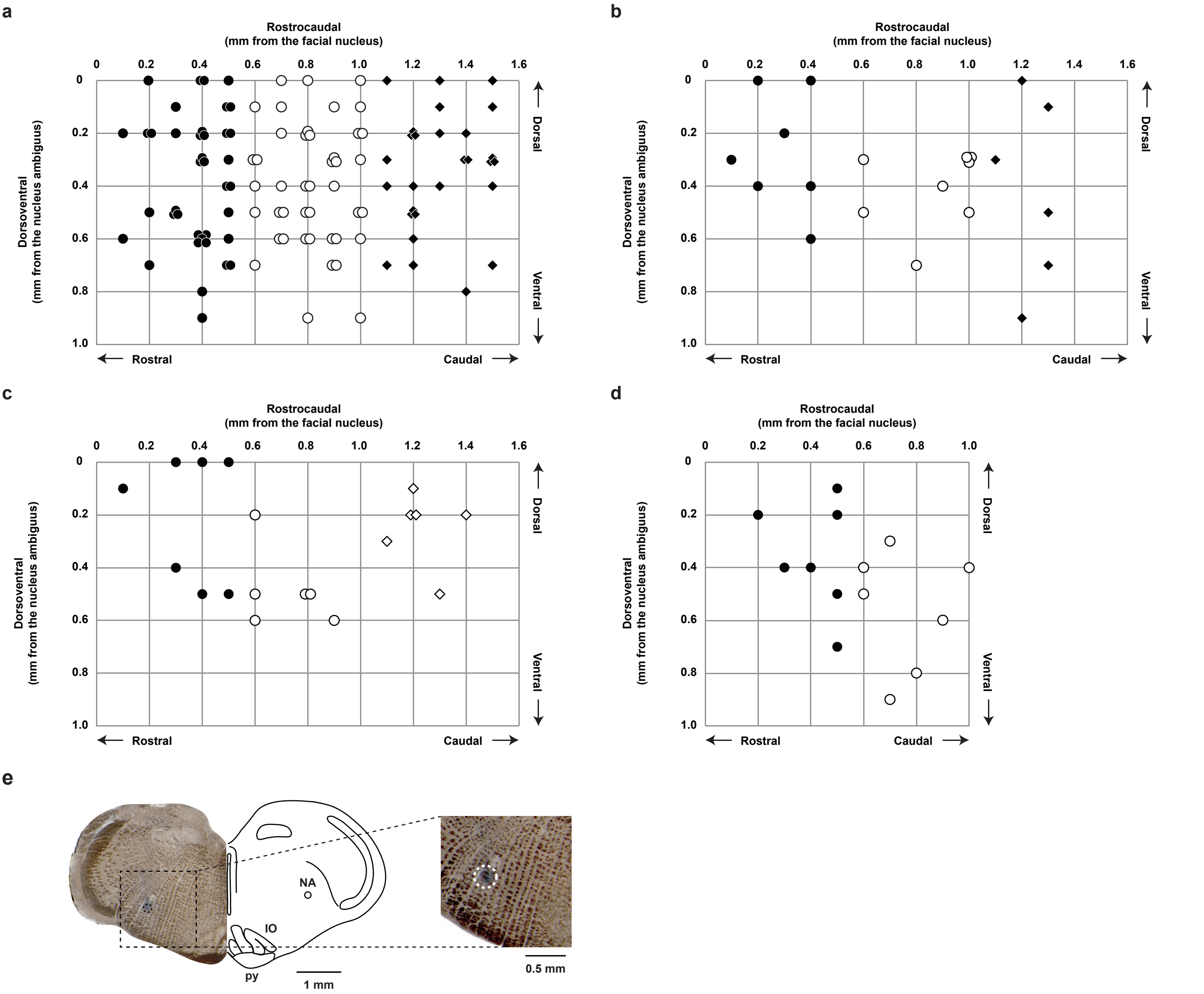

Figure S3.

Location of the injected sites

(a) Depth and rostrocaudal location of the center of all HA-injected sites in the BötC (black circle, n = 39), preBötC (open circle, n = 43), and rVRG (black diamond, n = 28). The depth of the injected sites was expressed as the distance from the ventral edge of the nucleus ambiguus. The rostrocaudal location was expressed as the distance from the caudal edge of the facial nucleus. **(b)** Depth and rostrocaudal location of the center of all KYN-injected sites in the BötC (black circle, n = 7), preBötC (open circle, n = 8), and rVRG (black diamond, n = 6). **(c)** Depth and rostrocaudal location of the center of all B+S-injected sites in the BötC (black circle, n = 7), preBötC (open circle, n = 6), and rVRG (black diamond, n = 6). **(d)** Depth and rostrocaudal location of the center of all KYN+HA-injected sites in the BötC (black circle, n = 7) and preBötC (open circle, n = 7). **(e)** Sagittal section of the medulla. The dotted circle indicates the pontamine-injected site. NA, nucleus ambiguus; IO, inferior olive; py, pyramidal tract.

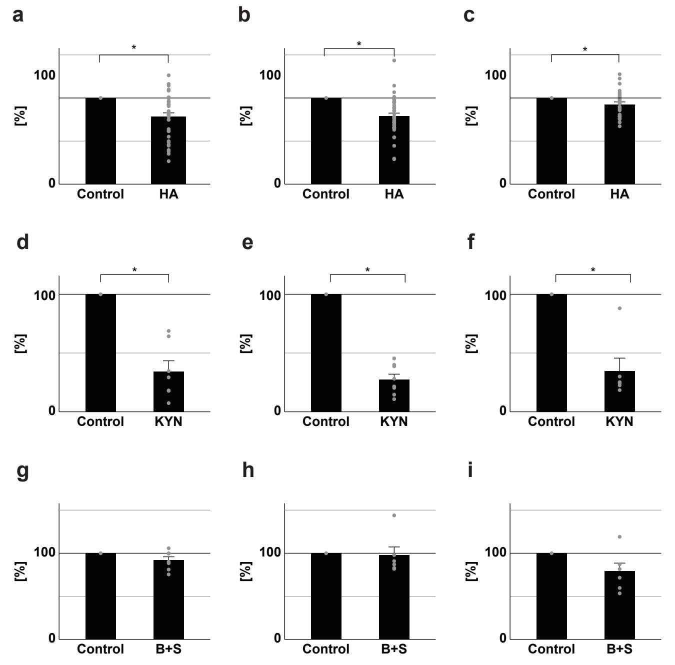

Figure S4.

Effects of H_2S synthesis inhibition on the amplitude of post-inspiratory vagus nerve activity

(a), (b), (c) Change rates of the amplitude of the post-inspiratory integrated activities of the phrenic nerve (∫PNA) after HA microinjection at the BötC (a), preBötC (b), and rVRG (c). **(d), (e), (f)** Change rates of the amplitude of the post-inspiratory integrated activities of the phrenic nerve (∫PNA) after KYN microinjection at the BötC (d), preBötC (e), and rVRG (f). **(g), (h), (i)** Change rates of the amplitude of the post-inspiratory integrated activities of the phrenic nerve (∫PNA) after B+S microinjection at the BötC (g), preBötC (h), and rVRG (i). The asterisks indicate $p < 0.05$ as compared with the control (one sample t-test). Results are expressed as means ± SEMs.
